# Effect of dapagliflozin according to baseline systolic blood pressure in the Dapagliflozin and Prevention of Adverse Outcomes in Heart Failure trial (DAPA-HF)

**DOI:** 10.1093/eurheartj/ehaa496

**Published:** 2020-08-21

**Authors:** Matteo Serenelli, Michael Böhm, Silvio E Inzucchi, Lars Køber, Mikhail N Kosiborod, Felipe A Martinez, Piotr Ponikowski, Marc S Sabatine, Scott D Solomon, David L DeMets, Olof Bengtsson, Mikaela Sjöstrand, Anna Maria Langkilde, Inder S Anand, Chern-En Chiang, Vijay K Chopra, Rudolf A de Boer, Mirta Diez, Andrej Dukát, Junbo Ge, Jonathan G Howlett, Tzvetana Katova, Masafumi Kitakaze, Charlotta E A Ljungman, Subodh Verma, Kieran F Docherty, Pardeep S Jhund, John J V McMurray

**Affiliations:** British Heart Foundation Cardiovascular Research Centre, University of Glasgow, 126 University Place, Glasgow G12 8TA, UK; Cardiovascular Institute, Azienda Ospedaliero-Universitaria di Ferrara, Cona, Italy; Klinik für Innere Medizin III, Universität des Saarlandes, Universitätsklinikum des Saarlandes, Homburg/Saar, Germany; Section of Endocrinology, Yale University School of Medicine, New Haven, CT, USA; Department of Cardiology, Rigshospitalet Copenhagen University Hospital, Copenhagen, Denmark; Saint Luke’s Mid America Heart Institute and University of Missouri-Kansas City, Kansas City, MO, USA; National University of Cordoba, Cordoba, Argentina; Department of Heart Diseases, Wroclaw Medical University, Wroclaw, Poland; Cardiovascular Division, Brigham and Women’s Hospital, Boston, MA, USA; Cardiovascular Division, Brigham and Women’s Hospital, Boston, MA, USA; Department of Biostatistics & Medical Informatics, University of Wisconsin, Madison, WI, USA; AstraZeneca R&D, Gothenburg, Sweden; AstraZeneca R&D, Gothenburg, Sweden; AstraZeneca R&D, Gothenburg, Sweden; Department of Cardiology, University of Minnesota, Minneaspolis, MN, USA; General Clinical Research Center and Division of Cardiology, Taipei Veterans General Hospital and National Yang-Ming University, Taipei, Taiwan; Department of Cardiology, Max Super Speciality Hospital, Saket, New Delhi, India; Department of Cardiology, University Medical Center and University of Groningen, Groningen, Netherlands; Division of Cardiology, Institute Cardiovascular de Buenos Aires, Buenos Aires, Argentina; Department of Internal Medicine, Comenius University in Bratislava, Bratislava, Slovakia; Department of Cardiology, Shanghai Institute of Cardiovascular Disease and Zhongshan Hospital Fudan University, Shanghai, China; Cardiac Sciences and Medicine, University of Calgary, Calgary, AB, Canada; Clinic of Cardiology, National Cardiology Hospital, Sofia, Bulgaria; Cardiovascular Division of Medicine, National Cerebral and Cardiovascular Center, Osaka, Japan; Department of Molecular and Clinical Medicine and Cardiology, Sahlgrenska Academy, Gothenburg, Sweden; Division of Cardiac Surgery, St Michael’s Hospital, University of Toronto, ON, Canada; British Heart Foundation Cardiovascular Research Centre, University of Glasgow, 126 University Place, Glasgow G12 8TA, UK; British Heart Foundation Cardiovascular Research Centre, University of Glasgow, 126 University Place, Glasgow G12 8TA, UK; British Heart Foundation Cardiovascular Research Centre, University of Glasgow, 126 University Place, Glasgow G12 8TA, UK

**Keywords:** Heart failure, Blood pressure, Hypotension, SGLT2 inhibitor

## Abstract

**Aims:**

Concern about hypotension often leads to withholding of beneficial therapy in patients with heart failure and reduced ejection fraction (HFrEF). We evaluated the efficacy and safety of dapagliflozin, which lowers systolic blood pressure (SBP),according to baseline SBP in Dapagliflozin and Prevention of Adverse Outcomes in Heart Failure trial (DAPA-HF).

**Methods and results:**

Key inclusion criteria were: New York Heart Association Class II−IV, left ventricular ejection fraction ≤ 40%, elevated N-terminal pro-B-type natriuretic peptide level, and SBP ≥95 mmHg. The primary outcome was a composite of worsening heart failure or cardiovascular death. The efficacy and safety of dapagliflozin were examined using SBP as both a categorical and continuous variable. A total of 1205 patients had a baseline SBP <110 mmHg; 981 ≥ 110 < 120; 1149 ≥ 120 < 130; and 1409 ≥ 130 mmHg. The placebo-corrected reduction in SBP from baseline to 2 weeks with dapagliflozin was −2.54 (−3.33 to −1.76) mmHg (*P *< 0.001), with a smaller between-treatment difference in patients in the lowest compared to highest SBP category. Patients in the lowest SBP category had a much higher rate (per 100 person-years) of the primary outcome [20.6, 95% confidence interval (95% CI) 17.6–24.2] than those in the highest SBP category (13.8, 11.7–16.4). The benefit and safety of dapagliflozin was consistent across the range of SBP; hazard ratio (95% CI) in each SBP group, lowest to highest: 0.76 (0.60–0.97), 0.76 (0.57–1.02), 0.81 (0.61–1.08), and 0.67 (0.51–0.87), *P* interaction = 0.78. Study drug discontinuation did not differ between dapagliflozin and placebo across the SBP categories examined.

**Conclusion:**

Dapagliflozin had a small effect on SBP in patients with HFrEF and was superior to placebo in improving outcomes, and well tolerated, across the range of SBP included in DAPA-HF.

**Clinical Trial Registration:**

ClinicalTrials.gov NCT03036124.

**Figure:**
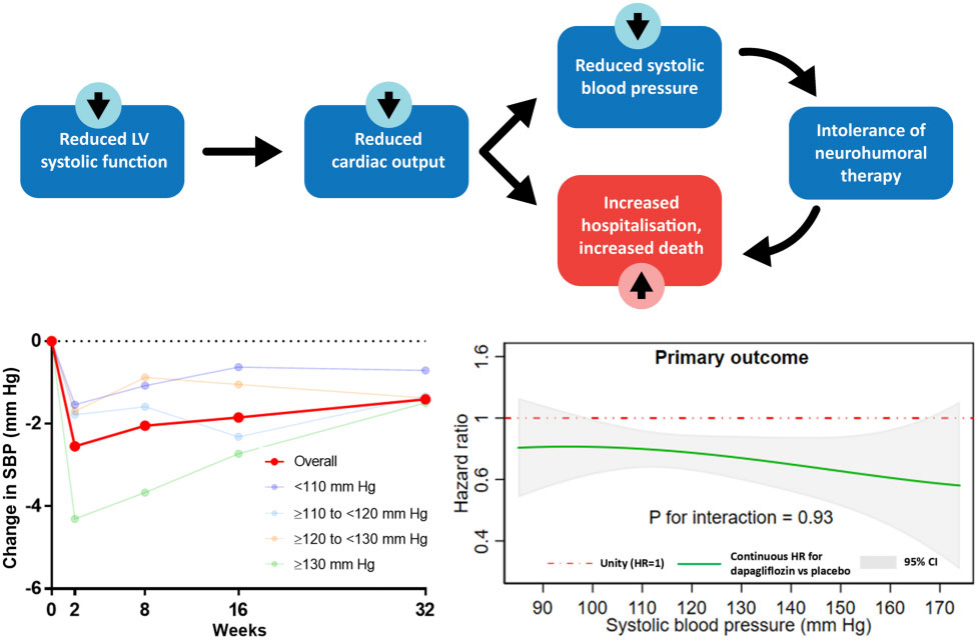

**See page 3419 for the editorial comment on this article (doi: 10.1093/eurheartj/ehaa584)**

## Introduction

The relationships between blood pressure, outcomes and the effects of treatment in patients with heart failure and reduced ejection fraction (HFrEF) have been described as paradoxical.^1–3^ Although most beneficial treatments for HFrEF reduce systolic blood pressure (SBP), HFrEF patients with lower SBP have worse outcomes than those with a higher SBP.^1–11^ These poor outcomes are often attributed to low cardiac output and worse haemodynamic status in patients with low SBP. However, the poor prognosis in patients with low SBP may also be due to underutilization of effective therapies.^12^ ^,^ ^13^ Underuse of these treatments reflects reluctance of physicians to prescribe agents perceived to precipitate or worsen hypotension and cause problems such as dizziness, syncope, and renal dysfunction.^12^ ^,^ ^13^ Consequently, it is essential that the effects of new treatments for HFrEF on SBP, and according to SBP, are fully understood. Sodium-glucose cotransporter-2 (SGLT2) inhibitors are recommended to lower risk of HF hospitalization in patients with diabetes and have been shown to reduce SBP in type 2 diabetic patients by 3–4 mmHg, similar in size to the reduction obtained with a low dose of a thiazide diuretic.^14^ ^,^ ^15^ We evaluated the effect of SGLT2 inhibition on SBP in HFrEF patients, both with and without diabetes, and the efficacy and safety of dapagliflozin according to baseline SBP, in the Dapagliflozin and Prevention of Adverse Outcomes in Heart Failure trial (DAPA-HF).^16–18^

## Methods

Dapagliflozin and Prevention of Adverse Outcomes in Heart Failure trial was a randomized, double-blind, placebo-controlled, event-driven, trial in patients with HFrEF. The efficacy and safety of dapagliflozin 10 mg once daily, added to standard care, was compared with matching placebo. The design, baseline characteristics, and primary results of the trial have been published.^16–18^ The Ethical Committee of each of the 410 participating institutions (in 20 countries) approved the protocol, and all patients gave written informed consent. The corresponding author had full access to the trial data and takes responsibility for its integrity and the data analysis. The data underlying this article were provided by AstraZeneca. Data will be shared on request to the corresponding author with permission of AstraZeneca.

### Study patients

Men and women aged ≥18 years with HF were eligible if they were in New York Heart Association (NYHA) functional Class II to IV, had a left ventricular ejection fraction (LVEF) ≤40%, and were optimally treated with pharmacological and device therapy for HF. Participants were also required to have a N-terminal pro-B-type natriuretic peptide (NT-proBNP) concentration ≥600 pg/mL (≥400 pg/mL if hospitalized for HF within the previous 12 months). Patients with atrial fibrillation or atrial flutter were required to have a NT-proBNP level ≥900 pg/mL, irrespective of history of HF hospitalization.Key exclusion criteria included: symptoms of hypotension or SBP <95 mmHg, estimated glomerular filtration rate (eGFR) <30 mL/min/1.73m^2^ (or rapidly declining renal function), and type 1 diabetes mellitus. A full list of exclusion criteria is provided in the design paper.^16^

### Study procedures

After the provision of informed consent, Visit 1 started a 14-day screening period during which the trial inclusion and exclusion criteria were checked, and baseline information were collected. Visit 2 was the randomization visit, and randomization was stratified based on diagnosis of type 2 diabetes at screening. After randomization, follow-up visits took place at 14 and 60 days, and then at 120, 240, 360 days, and every 4 months thereafter. The visit early after randomization (14 days) was included to check renal function and blood pressure (as well as for symptoms of hypotension); this visit also allowed for adjustment of background diuretic or other non-essential therapies. Dose reduction to 5 mg of dapagliflozin or matching placebo (or discontinuation of study drug) was to be considered in case of an acute unexpected decline in eGFR, volume depletion or hypotension (or to avoid these conditions); however, dose up-titration (or re-initiation) wasencouraged thereafter in all cases,where possible.

### Study outcomes

The primary outcome was the composite of an episode of worsening heart failure (HF hospitalization or an urgent visit because of worsening HF requiring intravenous therapy) or cardiovascular (CV) death, whichever occurred first. Secondary endpoints were the occurrence of HF hospitalization or CV death; HF hospitalizations (first and recurrent) and cardiovascular deaths; change from baseline to 8 months in the total symptom score of the Kansas City Cardiomyopathy Questionnaire (KCCQ-TSS);^19^ the incidence of a composite worsening renal function outcome, consisting of (a) ≥50% sustained decline in eGFR, (b) end-stage renal disease (defined as sustained eGFR < 15 mL/min/1.73 m^2^, chronic dialysis treatment or renal transplantation), or (c) renal death; and death from any cause. Because of the small number of renal events overall, this endpoint was not examined in the present analysis of subgroups. Prespecified safety analyses included any serious adverse event, adverse events leading to discontinuation of trial treatment, adverse events of interest (i.e. volume depletion, renal events, major hypoglycaemic events, bone fractures, diabetic ketoacidosis, and amputation), and any diagnosis of Fournier’s gangrene, as well as laboratory findings of note.

### Statistical analysis

In the present study, patients were divided into four baseline SBP categories, as in previous studies: (i) <110 mmHg, (ii) ≥110 to <120 mmHg, (iii) ≥120 to <130 mmHg, and (iv) ≥ 130 mmHg.^4–9^ ^,^ ^20^ Systolic blood pressure was measured at each trial visit (at 14, 60, 120, 240, and 360 days and every 4 months thereafter). Baseline characteristics were summarized as means and standard deviations (SDs), median and interquartile ranges, or percentages. Time-to-event data were evaluated with the use of the Kaplan–Meier estimates and Cox proportional-hazards models, stratified according to diabetes status, with a history of HF hospitalization and treatment group assignment as fixed-effect factors (as prespecified in the trial statistical analysis plan). In order to investigate a potentially non-linear relationship of risk across the spectrum of SBP, we also carried out fractional polynomial analyses of the association between SBP and the outcomes of interest. We used Cox models to calculate hazard ratios (HRs), 95% confidence intervals (CIs), and two-sided *P*-values and used a semiparametric proportional-rates model to calculate total (including recurrent) events, as previously described.^21^ We analysed the change in KCCQ-TSS from baseline to 8 months in surviving patients. Changes in SBP were assessed by the use of repeated measures mixed model with treatment, time, and treatment by time interaction as fixed effects, and time as random effect. Safety analyses were performed in patients who had undergone randomization and received at least one dose of dapagliflozin or placebo (a total of 8 out of 4744 patients were excluded). The effect of dapagliflozin compared with placebo on each outcome was also examined across the spectrum of blood pressure, in a Cox regression model in which SBP was modelled as a continuous variable. A fractional polynomial was constructed using SBP and entered into the model as an interaction term with treatment. The results of the interaction were displayed graphically using the ‘mfpi’ command in Stata. The polynomial allows for the possibility of a non-linear effect of treatment by blood pressure to be modelled. The interaction between SBP and treatment effect on the occurrence of the prespecified safety outcomes was tested in a logistic regression model with an interaction term between baseline SBP and treatment. The same analysis was performed for diastolic blood pressure (DBP) and pulse pressure (Supplementary material online, *Appendix*). The effect of differences in baseline characteristics was examined by adjustment of the model in sensitivity analyses (Supplementary material online, *Appendix*). Other sensitivity analyses took account of baseline and post-randomization SBP updated to the time of an event and time-updated SBP group (Supplementary material online, *Appendix*), the effect of treatment according to baseline DBP and the effect of treatment stratified by diabetes, history of hypertension and heart failure aetiology. As reported in another recent study,^8^ we also analysed outcomes in patients according to achieved SBP in each treatment category, with patients allocated to two categories according to their achieved SBP at 2 months (‘high’ or ‘low’) or four categories according to their starting *and* 2-month achieved SBP (high/high, high/low, low/high and, low/low with ‘low’ defined as ≤110 mm Hg and ‘high’ >110 mmHg) (Supplementary material online, *Appendix*). The correlation between baseline blood pressure and LVEF was studied analysing Pearson’s correlation coefficients (Supplementary material online, *Appendix*). The relationship between change in blood pressure with dapagliflozin at 2 weeks and baseline LVEF was examined by the use of fractional polynomial analysis (Supplementary material online, *Appendix*).

All analyses were conducted using Stata version 15.1 (College Station, TX, USA). A *P*-value < 0.05 was considered statistically significant.

## Results

The mean and median SBP in the 4744 patients randomized were 121.8 (SD 16.3) and 121.0 (Q1, Q3 109.7–132.0) mmHg, respectively. There were 1205 (25.4%) patients with a baseline SBP <110 mmHg (mean SBP 102.5 ± 4.9 mmHg), 981 (20.7%) with an SBP ≥110 to <120 mmHg (mean SBP 114.7 ± 2.9 mmHg), 1149 (24.2%) with an SBP ≥120 to <130 mmHg (mean SBP 124.3 ± 2.9 mmHg), 1409 (29.7%) with an SBP ≥130 mmHg (mean SBP 141.3 ± 11.2 mmHg).

### Patient characteristics

The baseline characteristics according to SBP category are shown in *Table 1*. Patients with a lower SBP were younger, more often male and of Asian race. A smaller proportion had a history of hypertension, diabetes, or coronary heart disease, but they had worse renal function, lower mean LVEF, and a higher median NT-proBNP level. The correlation between LVEF and baseline SBP is shown in Supplementary material online, *Figure S1*. Patients in the lowest SBP category were least likely to be treated with an angiotensin-converting enzyme inhibitor or angiotensin receptor blocker (ARB) but more frequently received treatment with a diuretic, mineralocorticoid receptor antagonist (MRA), and digoxin. Although overall use of sacubitril/valsartan was infrequent, patients in the lowest SBP category were proportionately most likely to be treated with it. The use of beta-blocker at baseline was similar across SBP categories. Patients with lower SBP were more frequently treated with device therapy, i.e. implantable cardioverter-defibrillator (ICD) or cardiac resynchronization therapy (CRT). Median baseline KCCQ-TSS and NYHA functional class were similar across SBP categories.

**Table 1:** Baseline characteristics according to systolic blood pressure category

Variables	**<110 mmHg (** *n* = **1205)**	**≥110 to <120 mmHg (** *n* = **981)**	**≥120 to <130 mmHg (** *n* = **1149)**	**≥130 (** *n* = **1409)**	*P*-value for trend
Systolic blood pressure (mmHg)^a^	102.5 (4.9)	114.7 (2.9)	124.3 (2.9)	141.3 (11.2)	
Diastolic blood pressure (mmHg)	64.9 (7.3)	70.9 (7.6)	75.3 (7.9)	81.2 (10.2)	
Age (years)	64.4 (11.7)	65.9 (11.3)	67.0 (10.2)	67.7 (10.1)	**<0.001**
Female *n* (%)	258 (21.4)	211 (21.5)	268 (23.3)	372 (26.4)	**0.001**
Race *n* (%)					**<0.001**
White	710 (58.9)	688 (70.1)	871 (75.8)	1,064 (75.5)	
Black or African American	64 (5.3)	44 (4.5)	47 (4.1)	71 (5.0)	
Asian	404 (33.5)	237 (24.2)	221 (19.2)	254 (18.0)	
Other	27 (2.2)	12 (1.2)	10 (0.9)	20 (1.4)	
Region *n* (%)					0.88
North America	228 (18.9)	138 (14.1)	141 (12.3)	170 (12.1)	
South America	257 (21.3)	176 (17.9)	155 (13.5)	229 (16.3)	
Europe	327 (27.1)	433 (44.1)	633 (55.1)	761 (54.0)	
Asia/Pacific	393 (32.6)	234 (23.9)	220 (19.1)	249 (17.7)	
HR (b.p.m.)	71.5 (12.0)	71.4 (12.3)	71.5 (11.3)	71.6 (11.4)	0.42
BMI (kg/m^2^)	26.7 (5.6)	27.7 (5.7)	28.6 (6.0)	29.3 (6.0)	**<0.001**
Creatinine (μmol/L)	107.3 (32.6)	103.5 (28.7)	103.3 (29.6)	103.6 (30.1)	**0.005**
Creatinine (mg/dL)	1.21 (0.37)	1.17 (0.32)	1.17 (0.33)	1.17 (0.34)	**0.005**
Estimated GFR (mL/min/1.73 m^2^)	65.7 (20.3)	66.9 (20.0)	65.8 (18.7)	65.0 (18.7)	0.44
Estimated GFR <60 ml/min/1.73 m^2^—*n*/total *n* (%)	509/1205 (42.2)	379/980 (38.7)	452/1149 (39.4)	586/1408 (41.6)	0.88
Median NT-proBNP (pg/mL) (IQR)	1611.9 (931.0–3114.6)	1502.2 (886.0–2682.0)	1357.7 (828.0–2480.6)	1334.0 (790.7–2381.5)	**<0.001**
Glycated haemoglobin^b^ *n* (%)	7.3 (1.5)	7.4 (1.5)	7.4 (1.5)	7.4 (1.6)	0.30
Heart failure aetiology *n* (%)					<0.001
Ischaemic	579 (48.0)	552 (56.3)	696 (60.6)	847 (60.1)	
Non-ischaemic	524 (43.5)	340 (34.7)	378 (32.9)	445 (31.6)	
Unknown	102 (8.5)	89 (9.1)	75 (6.5)	117 (8.3)	
Ejection fraction (%)	28.8 (7.2)	30.2 (6.7)	31.8 (6.5)	32.9 (6.1)	**<0.001**
NYHA Class *n* (%)					0.56
II	837 (69.5)	638 (65.0)	776 (67.5)	952 (67.6)	
III	353 (29.3)	327 (33.3)	367 (31.9)	451 (32.0)	
IV	15 (1.2)	16 (1.6)	6 (0.5)	6 (0.4)	
Total KCCQ score at baseline (IQR)	77.1 (58.3–91.7)	78.1 (60.4–91.7)	78.1 (58.3–91.7)	77.1 (58.3–92.7)	0.82
Hypertension	693 (57.5)	685 (69.8)	914 (79.5)	1,230 (87.3)	**<0.001**
Type 2 diabetes	437 (36.3)	392 (40.0)	470 (40.9)	684 (48.5)	**<0.001**
Atrial fibrillation	461 (38.3)	351 (35.8)	457 (39.8)	549 (39.0)	0.36
Hospitalization for heart failure	566 (47.0)	475 (48.4)	530 (46.1)	680 (48.3)	0.74
Prior MI	496 (41.2)	467 (47.6)	536 (46.6)	593 (42.1)	0.84
Prior PCI	384 (31.9)	321 (32.7)	421 (36.6)	498 (35.3)	**0.021**
Prior CABG	182 (15.1)	172 (17.5)	194 (16.9)	251 (17.8)	0.103
ACE inhibitor	584 (48.5)	581 (59.2)	699 (60.8)	797 (56.6)	**<0.001**
ARB	296 (24.6)	236 (24.1)	319 (27.8)	456 (32.4)	**<0.001**
ARNI	230 (19.1)	102 (10.4)	81 (7.0)	95 (6.7)	**<0.001**
Diuretic	1,160 (96.3)	927 (94.5)	1,076 (93.6)	1,270 (90.1)	**<0.001**
Digitalis	266 (22.1)	196 (20.0)	202 (17.6)	223 (15.8)	**<0.001**
Beta-blocker	1,152 (95.6)	942 (96.0)	1,108 (96.4)	1,356 (96.2)	0.36
Mineralocorticoid antagonist	931 (77.3)	739 (75.3)	841 (73.2)	859 (61.0)	**<0.001**
Oral anticoagulant	537 (44.6)	399 (40.7)	497 (43.3)	536 (38.0)	**0.004**
Antiplatelet therapy	628 (52.1)	536 (54.6)	635 (55.3)	793 (56.3)	**0.035**
Statin	769 (63.8)	652 (66.5)	809 (70.4)	946 (67.1)	**0.027**
ICD	288 (23.9)	214 (21.8)	219 (19.1)	232 (16.5)	**<0.001**
CRT-D	111 (9.2)	54 (5.5)	66 (5.7)	58 (4.1)	**<0.001**
ICD or CRT-D	399 (33.1)	268 (27.3)	285 (24.8)	290 (20.6)	**<0.001**
CRT-D/CRT-P	133 (11.0)	68 (6.9)	78 (6.8)	75 (5.3)	**<0.001**
Diabetes mellitus treatment *n* (%)^c^					
	**<110 mmHg (*n* = 437)**	**≥110 to <120 mmHg (*n* = 392)**	**≥120 to <130 mmHg (*n* = 470)**	**≥130 mmHg (*n* = 684)**	***P*-value for trend**
Biguanide	211 (48.3)	198 (50.5)	250 (53.2)	357 (52.2)	0.17
Sulfonylurea	81 (18.5)	94 (24.0)	94 (20.0)	169 (24.7)	0.052
DPP-4 inhibitor	81 (18.5)	57 (14.5)	64 (13.6)	108 (15.8)	0.29
GLP-1 receptor agonist	6 (1.4)	6 (1.5)	2 (0.4)	7 (1.0)	0.36
Insulin	123 (28.1)	97 (24.7)	120 (25.5)	200 (29.2)	0.50

a Median (Q1, Q3) - SBP < 110:102.7 (99–106.7); SBP ≥110–120; 115 (112–117.3); SBP ≥ 120–130: 124 (121.7–126.7); and SBP ≥130: 138.3 (133.3–145.3).

b Glycated haemoglobin values are listed only for patients with diabetes.

c The numbers are relative to patients with type II diabetes history at baseline.

ACE, angiotensin-converting enzyme; ARB, angiotensin receptor blocker; ARNI, angiotensin receptor neprilysin inhibitor; BMI, body mass index; CABG, coronary artery bypass grafting; CRT-D, cardiac resynchronization therapy-defibrillator; CRT-P, cardiac resynchronization therapy-pacemaker; DPP-4, dipeptidyl peptidase-4; GFR, glomerular filtration rate; GLP-1, glucagon-like peptide 1; ICD, implantable cardioverter-defibrillator; KCCQ, Kansas City Cardiomyopathy Questionnaire; PCI, percutaneous coronary intervention; MI, myocardial infarction; NYHA, New York Heart Association

### Change in blood pressure

**Figure 1:**
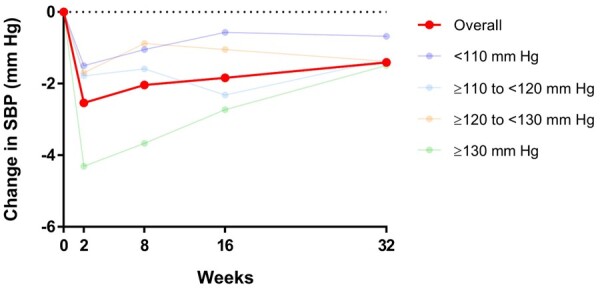
Placebo-corrected change in systolic blood pressure with dapagliflozin from baseline to 2 weeks, 2 months, 4 months, and 8 months. The figure shows effect of dapagliflozin on SBP during the first 8 months of treatment for the overall population and for each baseline SBP groups.

*Figure 1* summarizes the difference in change in SBP from baseline to 2 weeks, 2 months, 4 months, and 8 months in each treatment group and SBP category. These changes are also enumerated in *Table 2*. Overall, the mean change in SBP from baseline to 2 weeks was −0.5 (SD 12.0) mmHg in the placebo group and −3.1 (SD 12.3) mmHg in the dapagliflozin group, resulting in a between-treatment difference of −2.5 (95% CI −3.3 to −1.8; *P* < 0.001) mmHg. The corresponding values at 4 months were: −0.6 (SD 14.0) mmHg in the placebo group and −2.6 (SD 14.4) mmHg in the dapagliflozin group, difference −1.8 (95% CI −2.7 to −1.0; *P* < 0.001) mmHg. However, this overall mean change reflected a divergent pattern of change in patients with a lower and higher starting SBP. Specifically, SBP increased slightly in patients with the lowest baseline SBP (e.g. by 3.46 ± 10.21 in the placebo group and 1.91 ± 11.12 mmHg with dapagliflozin, at 2 weeks) and decreased in those starting with a higher baseline SBP (e.g. −4.62 ± 13.01 with placebo and −8.94 ± 13.26 mmHg with dapagliflozin, at the same time point in patients with SBP ≥130 mmHg). Nevertheless, SBP was still lower in patients assigned to dapagliflozin, compared with placebo, although the between-treatment difference was smaller in patients with the lowest baseline SBP (e.g. difference 1.50, 95% CI 0.09–2.92 mmHg at 2 weeks), compared with the highest SBP category (4.31, 95% CI 2.71–5.90 mmHg); *P* for interaction between baseline SBP category and effect of treatment on SBP was 0.012.

**Table 2:** Change in mean SBP from baseline to 2 weeks, 2, 4, and 8 months, and between-treatment difference in SBP, overall, and for each systolic blood pressure category

	Change in systolic blood pressure											
	Baseline to 2 weeks			Baseline to 2 months			Baseline to 4 months			Baseline to 8 months		
	Placebo	Dapa	Difference	Placebo	Dapa	Difference	Placebo	Dapa	Difference	Placebo	Dapa	Difference
All patients	−0.49 ± 11.95	−3.10 ± 12.33	−2.54 (−3.33 to −1.76) *P* < 0.001	−0.36 ± 13.30	−2.44 ± 13.66	−2.04 (−2.85 to −1.23) *P* < 0.001	−0.63 ± 13.95	−2.57 ± 14.44	−1.84 (−2.67 to −1.00) *P* < 0.001	−0.38 ± 15.27	−1.92 ± 14.92	−1.41 (−2.27 to −0.52) *P* = 0.002
SBP <110 mmHg	3.46 ± 10.21	1.91 ± 11.12	−1.50 (−2.92 to −0.09) = 0.037	4.26 ± 11.74	3.18 ± 12.27	−1.05 (−2.51 to 0.41) *P* = 0.16	4.49 ± 11.97	3.84 ± 13.40	−0.57 (−2.08 to 0.94) *P* = 0.46	6.05 ± 13.33	5.28 ± 12.97	−0.68 (−2.27 to 0.91) *P* = 0.40
SBP ≥110 to <120 mmHg	0.74 ± 10.51	−1.02 ± 10.63	−1.78 (−3.42 to −0.13) *P* = 0.034	1.34 ± 11.75	−0.23 ± 12.20	−1.59 (−3.28 to 0.09) *P* = 0.063	2.19 ± 13.38	−0.23 ± 12.38	−2.32 (−4.07 to −0.58) *P* = 0.009	2.73 ± 1.46	1.22 ± 12.81	−1.34 (−3.16 to 0.49) *P* = 0.15
SBP ≥120 to <130 mmHg	−0.84 ± 11.97	−2.59 ± 10.54	−1.70 (−3.23 to −0.16) *P* = 0.030	−0.66 ± 12.13	−1.56 ± 11.74	−0.88 (−2.44 to 0.69) *P* = 0.27	−1.33 ± 12.13	−2.38 ± 12.33	−1.05 (−2.65 to 0.56) *P* = 0.20	−1.25 ± 14.38	−2.53 ± 13.25	−1.38 (−3.04 to 0.28) *P* = 0.104
SBP ≥130 mmHg	−4.62 ± 13.01	−8.94 ± 13.26	−4.31 (−5.90 to −2.71) *P* < 0.001	−5.44 ± 14.80	−9.15 ±14.36	−3.67 (−5.30 to −2.05) *P* < 0.001	−6.57 ± 15.04	−9.34 ± 15.19	−2.73 (−4.39 to −1.07) *P* = 0.001	−7.48 ± 15.20	−9.03 ± 15.62	−1.49 (−3.21 to 0.24) *P* = 0.092

Dapa, dapagliflozin.

*P*-value for interaction between SBP groups and BP lowering effect over the duration of the trial = 0.012.

Of participants with a starting SBP ≥90 mmHg and with at least one SBP measurement during the first 8 months (*n* = 4691), 279 (5.9%) experienced a decrease in SBP below 90 mmHg; 131 (5.6%) in the placebo group and 148 (6.3%) in the dapagliflozin group (*P* = 0.32), without any interaction between SBP category and treatment (*P*-value for interaction = 0.61). Among participants with a baseline SBP ≥85 mmHg (*n* = 4697), 132 (2.8%) had a decrease in SBP to below 85 mmHg, 63 (2.7%) in the placebo group and 69 (2.9%) in the dapagliflozin group (*P* = 0.60), without any interaction between SBP category and treatment (*P-*value for interaction = 0.97).

The effect of dapagliflozin, compared with placebo, on DBP and pulse pressure is shown in the Supplementary material online, *Table S1* and *Figures S2* and *S3*; the overall pattern of response to dapagliflozin was similar to that seen for SBP. The effect of treatment on SBP and DBP according to aetiology of heart failure, history of hypertension, and diabetes status at baseline are also shown in the Supplementary material online, *Table S1*. The effect on both SBP and DBP were small in all subgroups examined. Because lower baseline blood pressure (and pulse pressure) was associated with lower LVEF, we also looked at the change in blood pressure (and pulse pressure) with dapagliflozin according to baseline LVEF. Systolic blood pressure and pulse pressure tended to increase in patients in the lowest LVEF category (Supplementary material online, *Figure S4*). We also examined change in background therapy and reduction in dose, withholding and discontinuation of study drug in each treatment group between baseline and 2 weeks (the blood pressure nadir). Overall, there were no important differences between the two treatment groups (Supplementary material online, *Tables S2–S4*).

### Association between systolic blood pressure and clinical outcomes and effect of dapagliflozin

**Figure 2:**
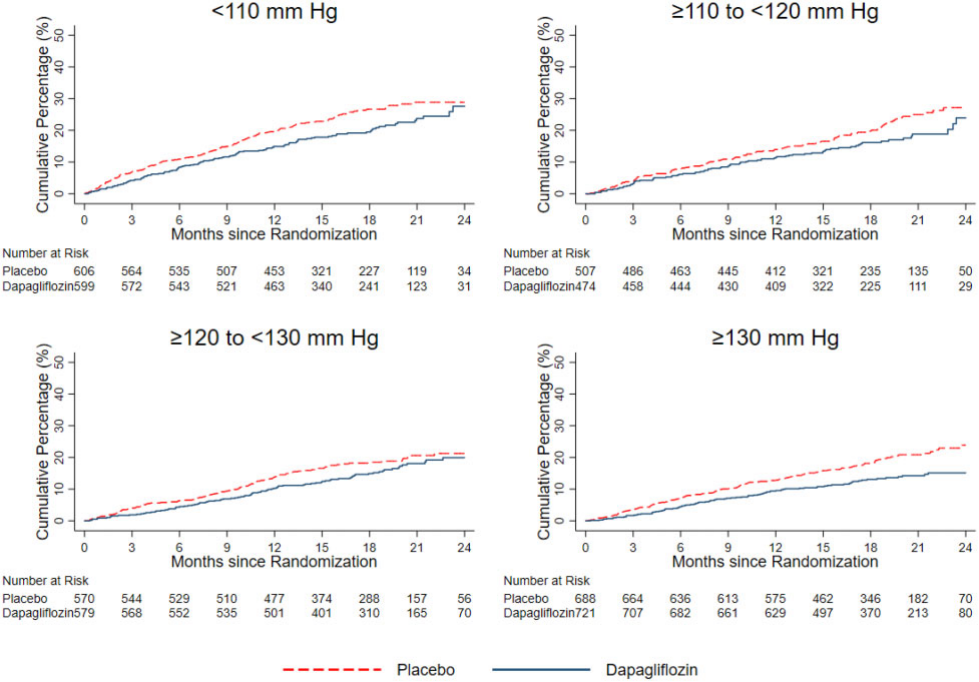
Cumulative Incidence of primary outcome in each systolic blood pressure category. The figure shows Kaplan–Meier event curves for placebo (red dashed line) and dapagliflozin (blue line) in each baseline SBP group.

The unadjusted incidences of the prespecified outcomes, according to baseline SBP, are shown in *Table 3, Figures 2* (primary outcome) and *3* (individual time-to-first death and hospitalization outcomes). Fractional polynomial analysis of the association between SBP and outcomes is shown in *Figure 4*.

**Figure 3:**
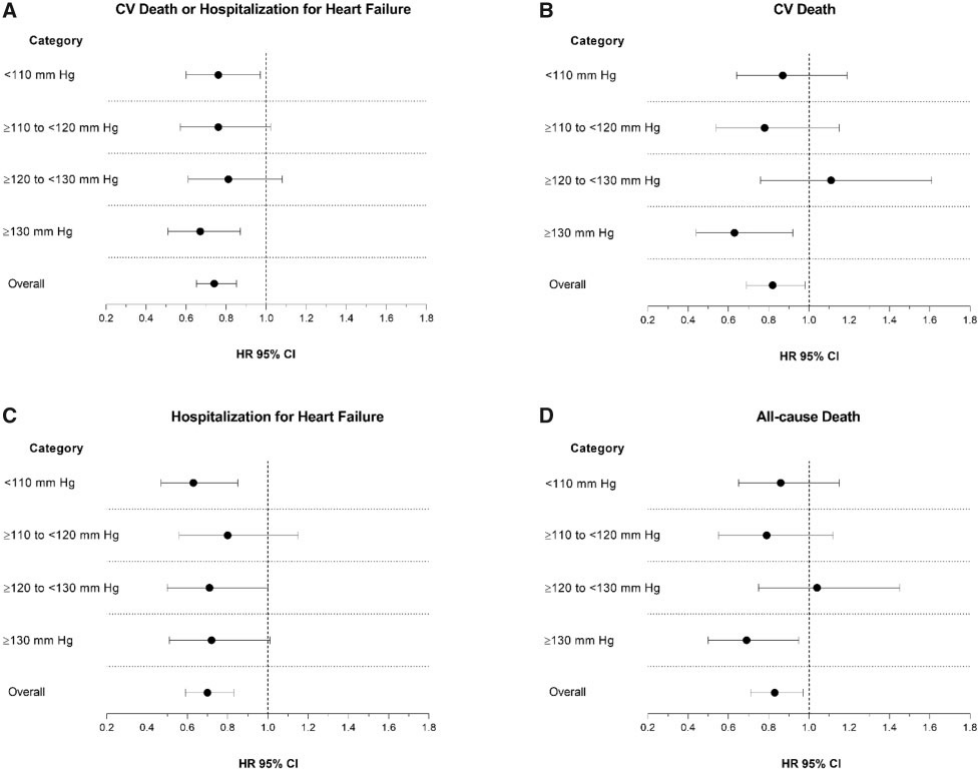
Hazard ratio for dapagliflozin, compared with placebo, for each outcome of interest, according to baseline systolic blood pressure category. The figures show unadjusted hazard ratios for the primary outcome (*A*), cardiovascular death (*B*), heart failure hospitalization/urgent visit (*C*), and all-cause death (*D*).

**Figure 4:**
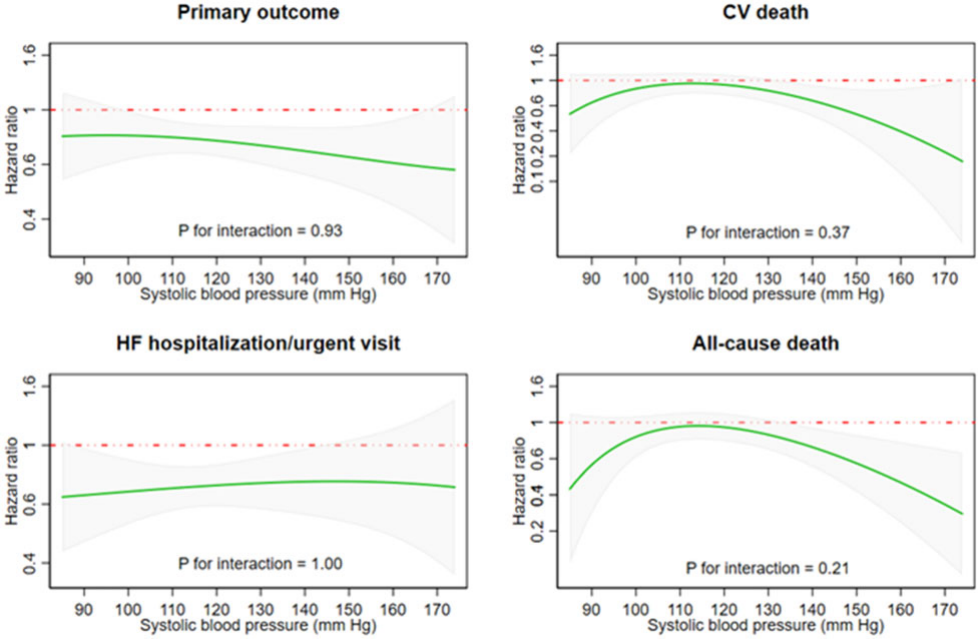
Hazard ratio for dapagliflozin, compared with placebo, for each clinical outcome, according to baseline systolic blood pressure modelled as a continuous variable. The figures show a continuous hazard ratio (green line) for treatment with dapagliflozin, compared to placebo, according to baseline systolic blood pressure. The interrupted red line shows a hazard ratio of 1 (i.e. unity, representing no treatment effect) and the grey shaded area the 95% confidence interval around the hazard ratio.

#### Primary outcome

The incidence of the primary composite outcome, in the placebo group, was highest in patients with the lowest SBP (<110 mmHg), next highest in those with SBP ≥110–<120 mmHg and plateaued in the SBP ≥120–<130 mmHg and SBP ≥130 mmHg groups.

The HR for the effect of dapagliflozin, compared with placebo, on the primary outcome, was consistent across the spectrum of SBP (*Table 3* and *Figure 3A*), and the *P*-value for interaction was 0.78.

**Table 3:** Clinical outcomes according to systolic blood pressure category

	<110 mmHg (*n* = 1205)		≥110 to <120 mmHg (*n* = 981)		≥120 to <130 mmHg (*n* = 1149)		≥130 mmHg *n* = 1409)		*P*-value for interaction
	Placebo (*n* = 606)	Dapagliflozin (*n* = 599)	Placebo (*n* = 507)	Dapagliflozin (*n* = 474)	Placebo (*n* = 570)	Dapagliflozin (*n* = 579)	Placebo (*n* = 688)	Dapagliflozin (*n* = 721)	
CV death or HF hospitalization/urgent HF visit									0.78
* n* (%)	155 (25.6)	122 (20.4)	109 (21.5)	79 (16.7)	106 (18.6)	92 (15.9)	132 (19.2)	93 (12.9)	
* *Rate (95% CI)	20.6 (17.6–24.2)	15.9 (13.3–19.0)	15.9 (13.2–19.2)	12.0 (9.7–15.0)	13.4 (11.1–16.3)	11.0 (9.0–13.6)	13.8 (11.7–16.4)	9.0 (7.4–11.1)	
* *Hazard ratio	0.76 (0.60–0.97)		0.76 (0.57–1.02)		0.81 (0.61–1.08)		0.67 (0.51–0.87)		
CV death									0.23
* n* (%)	87 (14.4)	76 (12.7)	63 (12.4)	46 (9.7)	52 (9.1)	58 (10.0)	71 (10.3)	47 (6.5)	
* *Rate (95% CI)	10.6 (8.6–13.1)	9.3 (7.5–11.7)	8.6 (6.7–11.0)	6.7 (5.0–8.9)	6.2 (4.7–8.1)	6.8 (5.2–8.7)	7.0 (5.6–8.9)	4.4 (3.3–5.8)	
* *Hazard ratio	0.87 (0.64–1.19)		0.78 (0.54–1.15)		1.11 (0.76–1.61)		0.63 (0.44–0.92)		
HF hospitalization/urgent HF visit									0.83
* n* (%)	109 (18.0)	72 (12.0)	69 (13.6)	52 (11.0)	72 (12.6)	55 (9.5)	76 (11.1)	58 (8.0)	
* *Rate (95% CI)	14.5 (12.0–17.5)	9.4 (7.4–11.8)	10.1 (8.0–12.8)	7.9 (6.0–10.4)	9.1 (7.2–11.5)	6.6 (5.1–8.6)	8.0 (6.4–10.0)	5.6 (4.4–7.3)	
* *Hazard ratio	0.63 (0.47–0.85)		0.80 (0.56–1.15)		0.71 (0.50–1.00)		0.72 (0.51–1.01)		
All-cause death									0.37
* n* (%)	102 (16.8)	88 (14.7)	73 (14.4)	54 (11.4)	68 (11.9)	72 (12.4)	86 (12.5)	62 (8.6)	
* *Rate (95% CI)	12.4 (10.2–15.1)	10.8 (8.8–13.3)	9.9 (7.9–12.5)	7.8 (6.0–10.2)	8.1 (6.4–10.3)	8.4 (6.7–10.6)	8.5 (6.9–10.5)	5.8 (4.5–7.4)	
* *Hazard ratio	0.86 (0.65–1.15)		0.79 (0.55–1.12)		1.04 (0.75–1.45)		0.69 (0.50–0.95)		
CV death and recurrent HF hospitalization									
* *No. of episodes	253	188	173	122	156	126	160	131	
* *Rate ratio	0.74 (0.56–0.97)		0.76 (0.54–1.06)		0.78 (0.58–1.06)		0.79 (0.59–1.05)		0.99
KCCQ total symptom score									
* *Placebo-corrected change in KCCQ-TSS score at 8 months with dapagliflozin	1.75 (−0.66 to 4.17)		4.92 (2.32 to 7.53)		0.64 (−1.73 to 3.02)		3.91 (1.72 to 6.10)		0.06
* *Patients with ≥5 points improvement in KCCQ at 8 months %	51.6	55.3	48.0	61.5	52.2	55.2	51.4	61.1	0.04
* *Patients with ≥5 points decrease in KCCQ at 8 months %	33.3	29.3	34.5	24.8	32.1	24.9	32.0	22.7	0.40

CV, cardiovascular; HF, heart failure; KCCQ, TSS Kansas City cardiomyopathy questionnaire total symptom score.

Applying the overall relative risk reduction (26%) to the placebo group event rate in those with SBP < 110 mmHg, gave an absolute risk reduction of 54 fewer patients experiencing a primary outcome per 1000 person-years of follow-up. The equivalent absolute risk reduction in patients with SBP ≥130 mmHg was estimated as 36 fewer patients per 1000 person-years of follow-up.

#### Cardiovascular death

The same pattern of relationship between SBP and rate of CV death was seen in the placebo group and participants in the lowest SBP category were at highest risk, as shown in *Table 3* and *Figure 3B*. The effect of dapagliflozin, compared with placebo, was consistent across the spectrum of SBP (*P*-value for interaction = 0.22).

#### Worsening heart failure events

There was a steeper gradient in worsening HF events across SBP categories than seen for CV death (*Table 3*). However, the effect of dapagliflozin, compared with placebo, remained consistent across SBP categories, including in patients with SBP <110 mmHg (*Table 3* and *Figure 3C*). Applying the overall relative risk reduction (30%) to the placebo group event rate in participants with SBP <110 mmHg, gave an absolute risk reduction of 32 per 1000 person-years of follow-up. The equivalent absolute risk reduction in patients with SBP ≥130 mmHg was 21 per 1000 person-years of follow-up.

#### All-cause mortality

The relationship between SBP and death from any cause was like the pattern seen for CV death. The effect of dapagliflozin compared with placebo was consistent across the spectrum of SBP (*Table 3* and *Figure 3D*; *P*-value for interaction 0.37). Applying the overall relative risk reduction (17%) to placebo group event rate in those with SBP <110 mmHg, gave an absolute risk reduction of 25 fewer deaths per 1000 person-years of follow-up. The equivalent absolute risk reduction in patients with SBP ≥130 mmHg was estimated as 14 fewer deaths per 1000 person-years.

#### Composite of recurrent heart failure hospitalization and cardiovascular death

As for the other endpoints, we observed a consistent effect of dapagliflozin on the occurrence of first and recurrent HF hospitalization and CV death across SBP categories (*Table 3*) (*P*-value for interaction =0.99).

### Effect of dapagliflozin compared to placebo examining systolic blood pressure as a continuous variable

*Figure 4* provides an alternative illustration of the effects of dapagliflozin compared with placebo, for the four outcomes described above, using fractional polynomial analysis. Each panel shows a continuous HR (with 95% CI) for dapagliflozin, compared with placebo, across the spectrum of SBP (SBP shown as a continuous variable on the X-axis). As in the categorical analysis, the effect of dapagliflozin, compared with placebo, was consistent across the entire spectrum of SBP, with non-significant *P*-values for interaction for all endpoints. Similar findings were also observed after adjusting for differences in baseline characteristics (Supplementary material online, *Table S5* and *Figure S5*).

The effect of dapagliflozin was also consistent across the range of DBP and pulse pressure included in the trial (Supplementary material online, *Figures S6* and *S7*). These findings were also true for both SBP and DBP, irrespective of aetiology of heart failure (Supplementary material online, *Figure S8a,b*) or history of hypertension (Supplementary material online, *Figure S9a,b*).

### Sensitivity analyses, time-updated systolic blood pressure analysis and achieved systolic blood pressure analysis

We also studied the effect of dapagliflozin in different Cox regression models taking account of, respectively: baseline SBP, baseline SBP category, baseline and post-randomization SBP updated to the time of an event, and time-updated SBP category (Supplementary material online, *Figures S10–S13*). These model adjustments did not change our finding of a consistent benefit of dapagliflozin, irrespective of baseline SBP.

Supplementary material online, *Figure S12* shows Kaplan–Meier curves for the achieved SBP at 2 months analysis (high or low category) and Supplementary material online, *Figure S13* the high/high, high/low, low/high, and low/low analysis of achieved SBP at 2 months (‘low’ defined as ≤110 mm Hg and ‘high’ >110 mmHg). While a low 2-month SBP was associated with worse outcomes in placebo-treated patients, this was not the case in those treated with dapagliflozin. In the 4-category analysis taking account of both baseline and 2-month SBP (high/high, high/low, low/high and low/low analysis), placebo-treated patients with persistently low SBP and those that decreased to low SBP after 2 months had worse outcomes than the equivalent dapagliflozin-treated patients (Supplementary material online, *Figure S13*).

### Change in Kansas City Cardiomyopathy Questionnaire at 8 months

As shown in *Table 3*, patients treated with dapagliflozin, overall, had a greater increase (improvement) in the KCCQ-TSS between baseline and 8 months and this benefit of dapagliflozin was consistent across SBP categories (*P*-value for interaction = 0.06). The proportion of patients with an improvement of KCCQ-TSS of ≥5 points was larger in patients treated with dapagliflozin, compared to patients treated with placebo. Conversely, the proportion of patients with a decrease in KCCQ-TSS of ≥5 points (i.e. a clinically meaningful deterioration) was smaller in those treated with dapagliflozin. The benefit of dapagliflozin over placebo in preventing deterioration of KCCQ-TSS, was consistent across SBP categories (*P*-value for interaction = 0.40). The proportion of participants reporting a ≥ 5-point improvement in KCCQ-TSS varied inconsistently across SBP categories, with an interaction between baseline SBP and treatment with dapagliflozin of borderline significance (*P*-value for interaction = 0.04).

### Prespecified safety assessments

The proportion of patients stopping study drug for any reason in the placebo group was highest in patients with the lowest SBP (*Table 4*). However, the rate of discontinuation was similar between dapagliflozin and placebo across all SBP categories (*P*-value for interaction =0.34). A similar pattern was seen for treatment discontinuation due to adverse events.

**Table 4:** Treatment discontinuation and adverse events according to systolic blood pressure category

SBP category	<110 mmHg (*n* = 1199)		≥110 to <120 mmHg (*n* = 981)		≥120 to <130 mmHg (*n* = 1149)		≥130 mmHg (*n* = 1407)		*P*-value for interaction^a^
	Placebo (*n* = 604)	Dapagliflozin (*n* = 595)	Placebo (*n* = 507)	Dapagliflozin (*n* = 474)	Placebo (*n* = 570)	Dapagliflozin (*n* = 579)	Placebo (*n* = 687)	Dapagliflozin (*n* = 720)	
Treatment discontinuation, *n* (%)									
Any reason	73 (12.1)	80 (13.4)	50 (9.9)	47 (9.9)	58 (10.2)	61 (10.5)	77 (11.2)	61 (8.5)	0.34
Adverse event	40 (6.6)	36 (6.1)	20 (3.9)	24 (5.1)	29 (5.1)	30 (5.2)	27 (3.9)	21 (2.9)	0.60
Adverse event, *n* (%)									
Volume depletion	74 (12.3)	79 (13.3)	31 (6.1)	37 (7.8)	25 (4.4)	27 (4.7)	32 (4.7)	35 (4.9)	0.93
Renal adverse event	50 (8.3)	44 (7.4)	40 (7.9)	23 (4.9)	44 (7.7)	30 (5.2)	36 (5.2)	56 (7.8)	**0.015**
Fracture	18 (3.0)	13 (2.2)	11 (2.2)	11 (2.3)	7 (1.2)	11 (1.9)	14 (2.0)	14 (1.9)	0.66
Amputation	5 (0.8)	1 (0.2)	1 (0.2)	2 (0.4)	2 (0.4)	4 (0.7)	4 (0.6)	6 (0.8)	0.31
Major hypoglycaemia	1 (0.2)	0 (0.0)	0 (0.0)	1 (0.2)	2 (0.4)	0 (0.0)	1 (0.1)	3 (0.4)	^c^
Leading to dose reduction	13 (2.2)	18 (3.0)	4 (0.8)	7 (1.5)	5 (0.9)	10 (1.7)	3 (0.4)	8 (1.1)	0.87
Serious adverse event, *n* (%)									
Any (including death)	298 (49.3)	255 (42.9)	212 (41.8)	191 (40.3)	214 (37.5)	201 (34.7)	270 (39.3)	248 (34.4)	0.69
Volume depletion	11 (1.8)	14 (2.4)	12 (2.4)	6 (1.3)	6 (1.1)	5 (0.9)	11 (1.6)	4 (0.6)	0.26
Renal serious adverse event	21 (3.5)	11 (1.8)	16 (3.2)	8 (1.7)	14 (2.5)	4 (0.7)	14 (2.0)	15 (2.1)	0.23
Renal function									
Mean change in creatinine to 8 months (mg/dL)^b^	0.03 (−0.00 to 0.06), *P* = 0.076		0.01 (−0.03 to 0.04), *P* = 0.61		0.03 (−0.00 to 0.05), *P* = 0.070		0.02 (−0.01 to 0.05), *P* = 0.26		0.77
Doubling of serum creatinine, *n* (%)	22 (3.6)	15 (2.5)	15 (3.0)	9 (1.9)	20 (3.5)	6 (1.0)	20 (2.9)	13 (1.8)	0.47

Patients receiving at least one dose of study drug.

a *P*-value is for interaction between systolic blood pressure category and the effect of treatment.

b Between-treatment difference in change from baseline to 8 months.

c *P*-value not provided because of few events.

Adverse events related to volume depletion were reported in 12.3% of the placebo group with SBP <110 mmHg and in 13.3% in the dapagliflozin group. *Serious* adverse events related to volume depletion occurred, overall, in 29 patients (1.2%) in the dapagliflozin group and 40 patients (1.7%) in the placebo group, with no interaction between SBP category and treatment (*P* for interaction = 0.26).

Renal adverse events were generally less frequent in patients treated with dapagliflozin than placebo for each SBP category, except for patients with SBP ≥130 mmHg who appeared to experience more renal adverse events with dapagliflozin (*P*-value for interaction = 0.015). However, *serious* renal events were less common with dapagliflozin, compared to placebo, across each SBP category (*P*-value for interaction = 0.23). The mean change in serum creatinine with dapagliflozin at 8 months was minimal across each SBP category (*P*-value for interaction =0.77) and relatively few patients in any SBP group (and either treatment group) experienced a doubling of serum creatinine.

## Discussion

We found that lower SBP was associated with worse outcomes in HFrEF, although risk increased steeply only in patients with SBP <110 mmHg, who constituted 25% of participants in DAPA-HF, in keeping with the proportion reported in recent registries.^1–9^ ^,^ ^12^ ^,^ ^20^ ^,^ ^22^ The benefit of dapagliflozin on death and hospitalization for heart failure was consistent across the range of SBP at baseline, whether SBP was analysed as a categorical or continuous variable (and the latter was also true for DBP). This remained true after adjustment for other baseline differences between patients in the various SBP categories and adjustment for SBP after randomization. Remarkably, compared with placebo, dapagliflozin was well tolerated in the lowest SBP group, despite reducing SBP slightly and even though patients with SBP <110 mmHg also had the worst renal function. Indeed, the rate of discontinuation of dapagliflozin was relatively low in participants with SBP <110 mmHg and not more than the rate of discontinuation of placebo (although the rate of discontinuation of both study treatments was slightly greater than in participants with a higher baseline SBP). Notably, patients in the lowest SBP group experienced an increase in SBP after randomization, while patients in the highest SBP group experienced a decrease. In part at least, this likely reflects the statistical phenomenon of ‘regression to the mean’, although SBP might also increase in some patients as a result of improvement in cardiac function with treatment.

Perhaps the most important finding of this study is that not only was dapagliflozin safe and well tolerated, even in patients with a baseline SBP <110 mmHg, but the absolute benefit of the drug was particularly large in those with the lowest SBP <110 mmHg. Indeed, because patients in the lowest SBP category had a higher rate of events, dapagliflozin-treated patients experienced 54 fewer primary outcomes per 1000 person-years of follow-up in this lowest SBP category compared with 36 fewer patients in the highest SBP category. Interestingly, patients in the lowest SBP group were well treated with conventional therapy, with only a slightly lower rate of use of renin–angiotensin system blockers (92% vs. 96% in the highest SBP category), a similar frequency of use of a beta-blocker and greater use of diuretic, digoxin, MRA, and sacubitril/valsartan, as well as cardiac resynchronization therapy and ICD. The greater use of the latter pharmacological and device therapies is likely to reflect more advanced disease in patients with a low SBP, as evidenced by their lower LVEF, higher NT-proBNP level and worse renal function. It is, therefore, important to emphasize that dapagliflozin has benefits over and above those of conventional disease-modifying therapies, especially in this highest risk group of patients. These findings should allay any concerns about using dapagliflozin in patients with low SBP.

It is also of interest to compare the effect of dapagliflozin on SBP in patients with HFrEF to its effect on SBP in patients without HFrEF. In a meta-analysis of 13 studies in individuals with type 2 diabetes, the placebo-corrected change in SBP from baseline to 6 months with dapagliflozin 10 mg was −3.6 (95% CI −4.9 to −2.4) mmHg, −2.6 (95% CI −3.4 to −1.8) mmHg, and −2.5 (95% CI −3.9 to −1.1) mmHg in patients with SBP >140 mm Hg, ≤140 mmHg, and ≤120 mmHg, respectively.^23^ In our patients with SBP ≥130 mmHg, the change at 2 weeks was −4.31 (95% CI −5.90 to −2.71) and −1.49 (95% CI −3.21 to 0.24) mmHg at 8 months. In participants with SBP <110 mmHg, there was a non-significant change of −1.50 (95% CI −2.92 to −0.09) mmHg at 2 weeks and −0.68 (95% CI −2.27 to 0.91) mmHg at 8 months. This finding of a smaller hypotensive effect of a blood pressure-lowering drug in HFrEF, compared to patients without HFrEF, is consistent with what has been found with beta-blockers, ARBs, and MRAs and remains unexplained.^4–9^ One hypothesis is that effective therapy may improve cardiac output in patients with HFrEF, offsetting any direct, treatment-induced, reduction in SBP.^8^ It is also notable that, in our supplementary analyses, a decrease in SBP in the placebo group was associated with worse outcomes, whereas that was not the case in the dapagliflozin group, emphasizing the prognostic difference between a spontaneous decline in SBP and one caused by the addition of a disease-modifying treatment.^4–9^

In view of the potential withholding of life-saving therapy due to concern about hypotension, it is also important to highlight that only a small proportion of patients experienced a decline in SBP to below 90 mmHg and this proportion was similar in each treatment group (6.3% with dapagliflozin and 5.6% with placebo); the equivalent proportion with a SBP decreasing to <85 mmHg was even smaller and balanced between treatment groups (2.9% and 2.7%, respectively).^1–3^ ^,^ ^12^ ^,^ ^22^ Likewise, no adverse event of interest was meaningfully more frequent with dapagliflozin, compared to placebo, in patients with SBP <110 mmHg.

Our analyses have some limitations. They are *post hoc* as no subgroup analysis was prespecified for the effect of treatment according to SBP (although analysis of change in SBP was prespecified). The SBP categories chosen were arbitrary (although the same as those used in prior studies).^4–9^ ^,^ ^20^ Our results are not applicable to patients with SBP <95 mmHg or presenting with symptoms of hypotension, as they were excluded from DAPA-HF.^16^ The other exclusion criteria (e.g. reduced eGFR) also limit the generalizability of our results.

In conclusion, dapagliflozin reduced the risk of death and worsening heart failure, and improved symptoms, across the broad range of baseline SBP studied in DAPA-HF. The effect of dapagliflozin on SBP was small in patients with HFrEF (*Take home figure*). There was no significant imbalance in adverse events or treatment discontinuation between dapagliflozin and placebo, even in individuals with SBP <110 mmHg.

**Take home figure:**
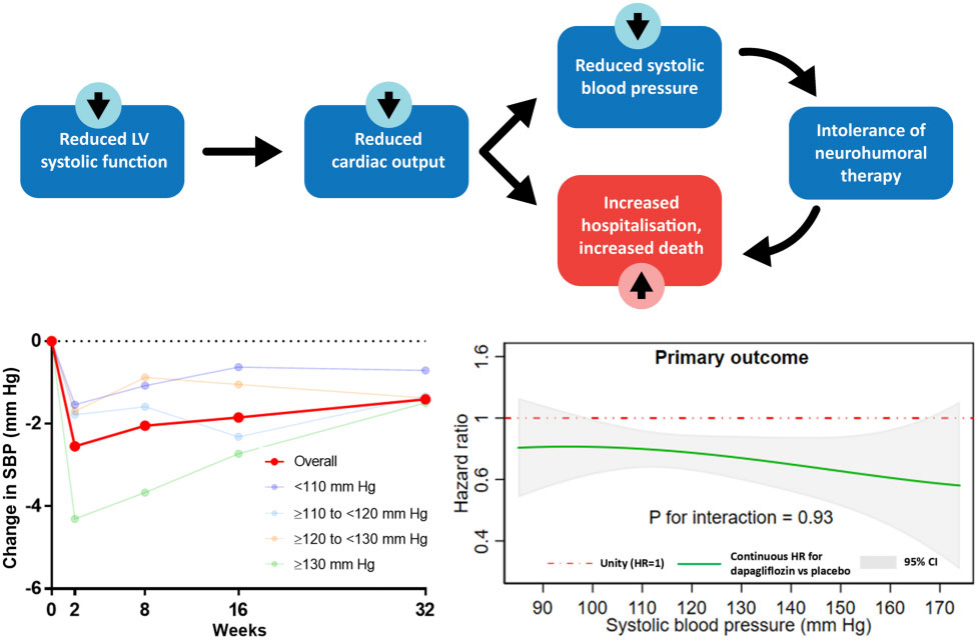
In patients with heart failure and impaired left ventricular (LV) systolic function, reduced cardiac output results in low systolic blood pressure (SBP) and heightened risk of adverse clinical outcomes. Hypotension also leads to withholding and intolerance of treatments that lower blood pressure further, denying patients life-saving therapy. We found that dapagliflozin resulted in a small reduction in systolic blood pressure andwas beneficial across the range of pressures measured at baseline in patients included in the Dapagliflozin and Prevention of Adverse Outcomes in Heart Failure trial (DAPA-HF).

## Funding

AstraZeneca; British Heart Foundation Centre of Research Excellence (RE/18/6/34217 to J.J.V.McM.).

**Conflict of interest:** K.F.D. reports receiving grant support from Novartis. P.S.J. reports his employer the University of Glasgow being paid by AstraZeneca for his time working on the DAPA-HF and DELIVER trials and by Novartis for working on the PARADIGM-HF and PARAGON-HF trials; receiving consulting fees, advisory board fees, and lecture fees from Novartis; advisory board fees from Cytokinetics; and grant support from Boehringer Ingelheim. S.E.I. receiving advisory fees from AstraZeneca and Zafgen; lecture fees, consulting fees, fees for serving as a clinical trial publications committee member, reimbursement for medical writing, and travel support from Boehringer Ingelheim; fees for serving on a steering committee and travel support from Sanofi/Lexicon; lecture fees, consulting fees, and travel support from Merck; and advisory fees and travel support from VTV Therapeutics and Abbott/Alere. L.K. receiving lecture fees from Novartis and Bristol-Myers Squibb. M.N.K. receiving grant support, honoraria, and research support from AstraZeneca; grant support and honoraria from Boehringer Ingelheim; and honoraria from Sanofi, Amgen, NovoNordisk, Merck (Diabetes), Eisai, Janssen, Bayer, GlaxoSmithKline, Glytec, Intarcia, Novartis, Applied Therapeutics, Amarin, and Eli Lilly. P.P. receiving consulting fees, fees for serving on a speakers bureau, and participating in clinical trials for Boehringer Ingelheim; lecture fees from Pfizer; participation in clinical trials for Amgen, grant support, paid to his institution; fees for serving on a speakers bureau, consulting fees, and participation in clinical trials for Vifor Pharma; fees for serving on a speakers bureau and consulting fees from Servier; fees for serving on a speakers bureau, consulting fees, and participation in clinical trials for Bayer; fees for serving on a speakers bureau, consulting fees, and participation in clinical trials for BMS; fees for serving on a speakers bureau and consulting fees from Respicardia; fees for serving on a speakers bureau from Berlin-Chemie; fees for serving on a speakers bureau, consulting fees, and participation in clinical trials for Cibiem; fees for serving on a speakers bureau, consulting fees, and participation in clinical trials for Novartis; and fees for serving on a speakers bureau, consulting fees, and participation in clinical trials for RenalGuard. D.L.D. receiving consulting fees from Frontier Science, Actelion, BMS, Medtronic, Boston Scientific, GSK, and Merck; and consulting fees and being owner of D L DeMets Consulting. M.S.S. receiving grant support, paid to Brigham and Women’s Hospital, and consulting fees from Amgen, AstraZeneca, Intarcia, Janssen Research and Development, The Medicines Company, MedImmune, Merck, and Novartis; consulting fees from Anthos Therapeutics, Bristol-Myers Squibb, CVS Caremark, DalCor, Dynamix, Esperion, IFM Therapeutics, and Ionis, grant support, paid to Brigham and Woman’s Hospital, from Bayer, Daiichi Sankyo, Eisai, GlaxoSmithKline, Pfizer, Poxel, Quark Pharmaceuticals, and Takeda; and serving as a member of the TIMI Study Group, which receives grant support, paid to Brigham and Women’s Hospital, from Abbott, Aralez, Roche, and Zora Biosciences. O.B. and M.S. being employed by AstraZeneca. A.M.L. being employed and holding shares in AstraZeneca. M.D. receiving personal fees from AstraZeneca during the conduct of the study. J.G.H. receiving grant support, consulting fees, and lecture fees from AstraZeneca, Boehringer Ingelheim, Novartis, and Servier; consulting fees and lecture fees from Novo Nordisk; consulting fees from Janssen, and grant support, consulting fees, lecture fees, and provision of drugs from Pfizer. T.K. receiving fees for serving as national coordinator of a trial from Novartis and AstraZeneca. C.E.A.L. receiving lecture fees and advisory board fees from AstraZeneca, lecture fees from Novartis, and advisory board fees from Pfizer. S.V. receiving grant support, lecture fees, and advisory board fees from AstraZeneca, Boehringer Ingelheim, Bayer, Janssen, and Merck; lecture fees from Sun Pharmaceuticals and EOCI Pharmacomm, grant support and advisory board fees from Amgen; and lecture fees and advisory board fees from Sanofi and Eli Lilly. S.D.S. receiving grant support and consulting fees from Alnylam, Amgen, AstraZeneca, BMS, Gilead, GSK, MyoKardia, Novartis, Theracos, Bayer, and Cytokinetics; grant support from Bellerophon, Celladon, Ionis, Lone Star Heart, Mesoblast, Sanofi Pasteur, and Eidos; consulting fees from Akros, Corvia, Ironwood, Merck, Roche, Takeda, Quantum Genomics, AoBiome, Caridac Dimensions, Tenaya, and Daiichi Sankyo; and fees for serving on a DSMB from Janssen. S.E.I reports personal fees and nonfinancial support from AstraZeneca, Boehringer Ingelheim, Sanofi/Lexicon, Merck, VTV Therapeutics, and Abbott/Alere, as well as personal fees from AstraZeneca and Zafgen. L.K reports other support from AstraZeneca and personal fees from Novartis and Bristol-Myers Squibb as a speaker. F.A.M reports personal fees from AstraZeneca. S.D.S reports grants from AstraZeneca, Bellerophon, Celladon, Ionis, Lone Star Heart, Mesoblast, National Institutes of Health/National Heart, Lung, and Blood Institute, Sanofi Pasteur, and Eidos; grants and personal fees from Alnylam, Amgen, AstraZeneca, BMS, Gilead, GSK, MyoKardia, Novartis, Theracos, Bayer, and Cytokinetics; and personal fees from Akros, Corvia, Ironwood, Merck, Roche, Takeda, Quantum Genomics, AoBiome, Janssen, Cardiac Dimensions, Tenaya, and Daichi-Sankyo. C.C. reports personal fees from AstraZeneca, Bayer, Boehringer Ingelheim, Daiichi-Sankyo, MSD, Novartis, Pfizer, and Sanofi. M.B. receiving lecture fees from Amgen, Bayer, Servier, Medtronic, Boehringer Ingelheim, Vifor Pharma, and Bristol-Myers Squibb, grant support and lecture fees from AstraZeneca, and grant support from Deutsche Forschungsgemeinschaft. I.S.A. receiving fees for serving as United States national leader of a trial from AstraZeneca, fees for serving on a steering committee from ARCA Biopharma, Amgen, LivaNova, and Novartis, fees for serving on an Endpoint committee from Boehringer Ingelheim, fees for serving as chair of a data and safety monitoring board from Boston Scientific, and advisory board fees from Zensun. R.A.B. grant support (paid to University Medical Center Groningen [UMCG]) and lecture fees from Novartis; grant support (paid to UMCG) from NovoNordisk, receiving grant support (paid to UMCG), consulting fees, and lecture fees from AstraZeneca, grant support (paid to UMCG) from Bristol-Myers Squibb, grant support (paid to UMCG) and consulting fees from Abbott, grant support (paid to UMCG) and lecture fees from Roche, and consulting fees from MandalMed and being a minority shareholder in scPharmaceuticals. J.G.H, receiving grant support, consulting fees, and lecture fees from AstraZeneca, Boehringer Ingelheim, Novartis, and Servier, consulting fees and lecture fees from Novo Nordisk, consulting fees from Janssen, and grant support, consulting fees, lecture fees, and provision of drugs from Pfizer. M.K. receiving grant support and lecture fees from Astellas Pharma, Sanofi, Pfizer, Ono Pharmaceutical, Novartis, and Mitsubishi Tanabe Pharma, lecture fees from Daiichi Sankyo, Bayer, Boehringer Ingelheim, Kowa Pharmaceutical, Sawai Pharmaceutical, MSD, Shionogi, Kureha, Taisho Toyama Pharmaceutical, Takeda Pharmaceutical, and Toa Eiyo, and manuscript fees from Japan Medical Data Center. C.EA.L receiving lecture fees and advisory board fees from AstraZeneca, lecture fees from Novartis, and advisory board fees from Pfizer. J.J.V.M. receiving fees (all fees listed paid to Glasgow University) for serving on a steering committee from Bayer, fees for serving on a steering committee, fees for serving on an endpoint committee, and travel support from Cardiorentis, fees for serving on a steering committee and travel support from Amgen, fees for serving on a steering committee and travel support from Oxford University/Bayer, fees for serving as principal investigator of a trial and travel support from Theracos, fees for serving on a steering committee and travel support from AbbVie, fees for serving on a steering committee from DalCor, fees for serving on a data safety monitoring committee from Pfizer, fees for serving on a data safety monitoring committee from Merck, fees for serving on an executive committee, fees for serving as co-principal investigator of a trial, fees for serving on a steering committee, fees for serving on an executive committee, travel support, and advisory board fees from Novartis, fees for serving as co-principal investigator for a trial, fees for serving on a steering committee, and travel support from GlaxoSmithKline, fees for serving on a steering committee from Bristol-Myers Squibb, fees for serving on a steering committee, fees for serving on an endpoint adjudication committee, and travel support from Vifor-Fresenius. No other conflict of interest relevant to this article was reported.

## Supplementary Material

ehaa496_Supplementary_Tables_and_Figures
