# Supplementary material for: Effect of dapagliflozin according to baseline systolic blood pressure in the Dapagliflozin and Prevention of Adverse Outcomes in Heart Failure trial (DAPA-HF)
Source: Eur Heart J. 2020 Aug 21;41(36):3402–18. doi: 10.1093/eurheartj/ehaa496 (PMC7550197; doi:10.1093/eurheartj/ehaa496)

**Supplementary tables and figures.**

**Table S1.** **Between-treatment difference (placebo - dapagliflozin) in blood pressure over time.** Change in systolic and diastolic blood pressure (SBP and DBP) at 2 weeks and 2, 4 and 8 months after randomization overall and in certain patient subgroups: diabetes status at randomization, history of hypertension and aetiology (ischaemic vs. non-ischaemic/unknown).

|  | **Between-treatment difference (placebo - dapagliflozin) in blood pressure over time** | | | | | | | |
| --- | --- | --- | --- | --- | --- | --- | --- | --- |
|  | **Baseline to 2 weeks** | | **Baseline to 2 months** | | **Baseline to 4 months** | | **Baseline to 8 months** | |
|  | **SBP**  **difference** | **DBP difference** | **SBP**  **difference** | **DBP**  **difference** | **SBP**  **difference** | **DBP**  **difference** | **SBP**  **difference** | **DBP difference** |
| **All patients**  **(n=4744*)** | -2.54  (-3.33 to -1.76)  P<0.001 | -1.09  (-1.62 to -0.57)  P<0.001 | -2.04  (-2.85 to -1.23)  P<0.001 | -0.61  (-1.15 to -0.07)  P=0.026 | -1.84  (-2.67 to -1.00)  P<0.001 | -0.71  (-1.26 to -0.16)  P=0.012 | -1.41  (-2.27 to -0.52)  P=0.002 | -0.34  (-0.91 to 0.24)  P=0.25 |
| **Type 2 diabetes**  **(n=1983*)** | -3.06  (-4.32 to -1.79)  P<0.001 | -1.36  (-2.18 to -0.54)  P=0.001 | -2.81  (-4.10 to -1.51)  P<0.001 | -1.38  (-2.21 to -0.54)  P=0.001 | -1.75  (-3.08 to -0.41)  P=0.011 | -0.78  (-1.64 to 0.08)  P=0.075 | -1.24  (-2.64 to 0.16)  P=0.084 | -0.41  (-1.30 to 0.50)  P=0.38 |
| **No diabetes**  **(n=2761*)** | -2.18  (-3.18 to -1.17)  P<0.001 | -0.90  (-1.59 to -0.22)  P=0.009 | -1.49  (-2.53 to -0.46)  P=0.005 | -0.06  (-0.76 to 0.64)  P=0.87 | -1.91  (-2.97 to -0.84)  P<0.001 | -0.66  (-1.38 to 0.06)  P=0.074 | -1.51  (-2.63 to -0.41)  P=0.007 | -0.29  (-1.04 to 0.47)  P=0.46 |
| **History of hypertension**  **(n=3522*)** | -2.67  (-3.62 to -1.72)  P<0.001 | -1.24  (-1.86 to -0.62)  P<0.001 | -2.31  (-3.28 to -1.34)  P<0.001 | -0.72  (-1.35 to - 0.08)  P=0.027 | -1.97  (-2.97 to -0.97)  P<0.001 | -0.91  (-1.56 to -0.26)  P=0.006 | -1.25  (-2.30 to -0.20)  P=0.020 | -0.08  (-0.76 to 0.60)  P=0.82 |
| **No history of hypertension**  **(n=1222*)** | -2.18  (-3.57 to -0.79)  P=0.002 | -0.66  (-1.63 to 0.30)  P=0.18 | -1.29  (-2.72 to 0.13)  P=0.075 | -0.30  (-1.30 to 0.69)  P=0.55 | -1.48  (-2.95 to -0.02)  P=0.047 | -0.13  (-1.15 to 0.90)  P=0.81 | -1.86  (-3.40 to -0.33)  P=0.017 | -1.08  (-2.16 to -0.00)  P=0.05 |
| **Ischaemic aetiology**  **(n=2674*)** | -2.57  (-3.61 to -1.52)  P<0.001 | -0.96  (-1.63 to -0.29)  P=0.005 | -2.44  (-3.52 to -1.37)  P<0.001 | -0.69  (-1.37 to 0.00)  P=0.050 | -2.38  (-3.48 to -1.27)  P<0.001 | -0.83  (-1.54 to -0.13)  P=0.021 | -1.34  (-2.49 to -0.18)  P=0.024 | -0.25  (-0.99 to 0.48)  P=0.50 |
| **Non-ischaemic/ unknown aetiology**  **(n=2070*)** | -2.50  (-3.70 to -1.30)  P<0.001 | -1.27  (-2.10 to -0.44)  P=0.003 | -1.55  (-2.78 to -0.32)  P=0.013 | -0.52  (-1.37 to 0.33)  P=0.23 | -1.18  (-2.45 to 0.09)  P=0.069 | -0.56  (-1.44 to 0.32)  P=0.21 | -1.49  (-2.82 to -0.16)  P=0.028 | -0.44  (-1.36 to 0.47)  P=0.34 |

**Number of patients with at baseline*

**Table S2. Change in beta-blocker, ACEi. ARB and MRA dose**

|  | **Placebo** | **Dapagliflozin** | **P value** | **Odds ratio**  **(95% CI)** |
| --- | --- | --- | --- | --- |
| **Beta-blocker (n=4717)†** |  |  |  |  |
| Decrease | 26 (1.1) | 23 (1.0) | 0.67 | 0.88 (0.50-1.55) |
| Increase | 21 (0.9) | 23 (1.0) | 0.76 | 1.10 (0.60-1.99) |
| No change | 2311 (98.0) | 2313 (98.1) | 0.92 | 1.02 (0.68-1.54) |
| **ACE-inhibitor (n=4734)†** |  |  |  |  |
| Decrease | 19 (0.8) | 13 (0.6) | 0.29 | 0.68 (0.34-1.38) |
| Increase | 15 (0.6) | 5 (0.2) | 0.033 | 0.33 (0.12-0.91) |
| No change | 2331 (98.6) | 2351 (99.2) | 0.02 | 1.91 (1.07-3.38) |
| **Angiotensin receptor blocker (n=4730)†** |  |  |  |  |
| Decrease | 12 (0.5) | 15 (0.6) | 0.57 | 1.25 (0.58-2.68) |
| Increase | 9 (0.4) | 8 (0.3) | 0.81 | 0.89 (0.34-2.30) |
| No change | 2342 (99.1) | 2344 (99.0) | 0.77 | 0.91 (0.50-1.66) |
| **Mineralocorticoid receptor antagonist (n=4735)†** |  |  |  |  |
| Decrease | 21 (0.9) | 21 (0.9) | 1.00 | 1.00 (0.54-1.83) |
| Increase | 28 (1.2) | 12 (0.5) | 0.013 | 0.43 (0.22-0.84) |
| No change | 2317 (97.9) | 2336 (98.6) | 0.08 | 1.50 (0.96-2.34) |

*Percentages may not total to 100 due to rounding*

*† Number of patients with determinate dose at both time points (including patients not on medication)*

*Odd ratios presented for dapagliflozin versus placebo (referent).*

**Table S3. Change in loop diuretic dose**

|  | **Placebo** | **Dapagliflozin** | **P value** | **Odds ratio**  **(95% CI)** |
| --- | --- | --- | --- | --- |
| **Baseline** |  |  |  |  |
| Daily dose (n=3697)* |  |  |  |  |
| Median – mg  Mean – mg | 40 (20-80)  60.2±96.9 | 40 (20-80)  58.3±94.9 | 0.33  0.57 | -  - |
| **14 days (n=4595)†** |  |  |  |  |
| Daily dose (n=3688)* |  |  |  |  |
| Median – mg  Mean – mg | 40 (20-80)  60.3±97.5 | 40 (20-80)  58.7±97.0 | 0.40  0.62 |  |
| Decrease | 32 (1.4) | 35 (1.5) | 0.72 | 1.10 (0.67-1.78) |
| Increase | 40 (1.7) | 27 (1.2) | 0.11 | 0.67 (0.41-1.10) |
| No change | 2222 (96.9) | 2239 (97.3) | 0.37 | 1.17 (0.83-1.65) |

*Daily doses shown are only for those patients taking a loop diuretic.*

** Number of patients on a loop diuretic at given time point*

† *Number of patients with determinate loop diuretic dose at both time points (including patients on no loop diuretic)*

*furosemide 80mg PO = furosemide 40mg IV/SC = bumetanide 1mg = azosemide 60mg = torsamide 20mg = ethacrynic acid 100mg*

**Table S4. Study drug interruption, dose reduction and treatment discontinuation overall and at 14 days.**

|  | **Placebo**  **(n=2368)** | **Dapagliflozin**  **(n=2368)** | **Odds Ratio**  **(95% CI)** | **P-value** |
| --- | --- | --- | --- | --- |
| **Dose interruption overall** | 396 (16.7%) | 341 (14.4%) | 0.84 (0.72-0.98) | 0.028 |
| **Dose interruption at 14 days** | 20 (0.8%) | 30 (1.3%) | 1.51 (0.85-2.66) | 0.16 |
| **Dose reduction overall** | 39 (1.6%) | 45 (1.9%) | 1.16 (0.75-1.78) | 0.51 |
| **Dose reduction at 14 days** | 0 (0.0%) | 5 (0.2%) | - | 0.025 |
| **Permanent discontinuation overall** | 258 (10.9%) | 249 (10.5%) | 0.96 (0.80-1.16) | 0.67 |
| **Permanent discontinuation at 14 days** | 20 (0.8%) | 18 (0.8%) | 0.90 (0.47-1.70) | 0.75 |

**Table S5.** **Clinical outcomes according to systolic blood pressure categories.** Cox regression model is adjusted for age, gender, race, region, heart rate, BMI, history of hypertension, type II diabetes, atrial fibrillation, previous MI, PCI, CABG, stroke, etiology of heart failure, baseline eGFR, ejection fraction, NYHA class and NT-proBNP level.

|  | **<110 mmHg (n=1205)** | | | **≥110 to <120 mmHg (n=981)** | | | **≥120 to <130 mmHg (n=1149)** | | | **≥130 mmHg (n=1409)** | | | **P value for interaction** |
| --- | --- | --- | --- | --- | --- | --- | --- | --- | --- | --- | --- | --- | --- |
| **Outcome** | **Placebo**  **(n=606)** | **Dapagliflozin**  **(n=599)** | | **Placebo**  **(n=507)** | **Dapagliflozin**  **(n=474)** | | **Placebo**  **(n=570)** | **Dapagliflozin**  **(n=579)** | | **Placebo**  **(n=688)** | | **Dapagliflozin**  **(n=721)** |  |
| **CV death or HF hospitalization/urgent HF visit** |  |  | |  |  | |  |  | |  | |  | 0.76 |
| No. (%) | 155 (25.6) | 122 (20.4) | | 109 (21.5) | 79 (16.7) | | 106 (18.6) | 92 (15.9) | | 132 (19.2) | | 93 (12.9) |  |
| Rate (95% CI) | 20.6  (17.6-24.2) | 15.9  (13.3-19.0) | | 15.9  (13.2-19.2) | 12.0  (9.7-15.0) | | 13.4  (11.1-16.3) | 11.0  (9.0-13.6) | | 13.8  (11.7-16.4) | | 9.0  (7.4-11.1) |  |
| Hazard ratio | 0.78 (0.61-1.00), P=0.047 | | | 0.74 (0.55-1.00), P=0.051 | | | 0.85 (0.64-1.13), P=0.26 | | | 0.66 (0.50-0.86), P=0.002 | | |  |
| **CV death** |  |  | |  |  | |  |  | |  | |  | 0.13 |
| No. (%) | 87 (14.4) | 76 (12.7) | | 63 (12.4) | 46 (9.7) | | 52 (9.1) | 58 (10.2) | | 71 (10.3) | | 47 (7.5) |  |
| Rate (95% CI) | 10.6  (8.6-13.1) | 9.3  (7.5-11.7) | | 8.6  (6.7-11.0) | 6.7  (5.0-8.9) | | 6.2  (4.7-8.1) | 6.8  (5.2-8.7) | | 7.0  (5.6-8.9) | | 4.4  (3.3-5.8) |  |
| Hazard ratio | 0.95 (0.69-1.31), P=0.76 | | | 0.76 (0.51-1.13), P=0.18 | | | 1.22 (0.83-1.80), P=0.32 | | | 0.61 (0.42-0.89), P=0.011 | | |  |
| **HF hospitalization/urgent visit** |  |  | |  |  | |  |  | |  | |  | 0.81 |
| No. (%) | 109 (18.0) | 72 (12.0) | | 69 (13.6) | 52 (11.0) | | 72 (12.6) | 55 (9.5) | | 76 (11.1) | | 58 (8.0) |  |
| Rate (95% CI) | 14.5  (12.0-17.5) | 9.4  (7.4-11.8) | | 10.1  (8.0-12.8) | 7.9  (6.0-10.4) | | 9.1  (7.2-11.5) | 6.6  (5.1-8.6) | | 8.0  (6.4-10.0) | | 5.6  (4.4-7.3) |  |
| Hazard ratio | 0.62 (0.45-0.84), P=0.002 | | | 0.82 (0.57-1.19), P=0.30 | | | 0.73 (0.51-1.04), P=0.081 | | | 0.72 (0.51-1.02), P=0.065 | | |  |
| **All-cause death** |  | | |  | | |  | | |  | | | 0.23 |
| No. (%) | 102 (16.8) | | 88 (14.7) | 73 (14.4) | | 54 (11.4) | 68 (11.9) | | 72 (12.4) | 86 (12.5) | 62 (8.6) | |  |
| Rate (95% CI) | 12.4  (10.2-15.1) | | 10.8  (8.8-13.3) | 9.9  (7.9-12.5) | | 7.8  (6.0-10.2) | 8.1  (6.4-10.3) | | 8.4  (6.7-10.6) | 8.5  (6.9-10.5) | 5.8  (4.5-7.4) | |  |
| Hazard ratio | 0.94 (0.70-1.26), P=0.67 | | | 0.80 (0.55-1.14), P=0.22 | | | 1.15 (0.81-1.61), P=0.44 | | | 0.69 (0.49-0.96), P=0.028 | | |  |
| **CV death and recurrent HF hospitalization** |  | | |  | | |  | | |  | | | 0.98 |
| No. | 253 | | 188 | 173 | | 122 | 156 | | 126 | 160 | 131 | |  |
| Rate ratio | 0.76 (0.57-1.01), P=0.059 | | | 0.80 (0.57-1.12), P=0.20 | | | 0.84 (0.62-1.14), P=0.27 | | | 0.79 (0.59-1.07), P=0.13 | | |  |

CV denotes cardiovascular, and HF heart failure.

**Figure S1.** **Correlation between baseline (pre-randomization) LVEF and baseline (pre-randomization) blood pressure and pulse pressure (both treatment groups combined).** Red squares represent means, dashed lines represent ± 1 SD, grey areas represent 95% CI.


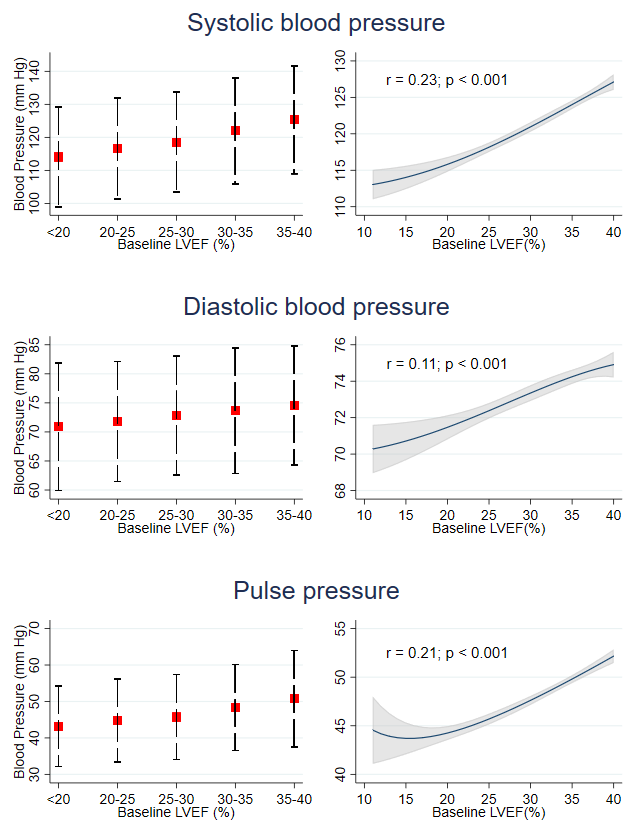


**FIGURE S2. Placebo-corrected change in diastolic blood pressure (DBP) with dapagliflozin from baseline to 2 weeks, 2 months, 4 months and 8 months.** The figure shows effect of dapagliflozin on DBP during the first 8 months of treatment for the overall population and for each baseline DBP groups.


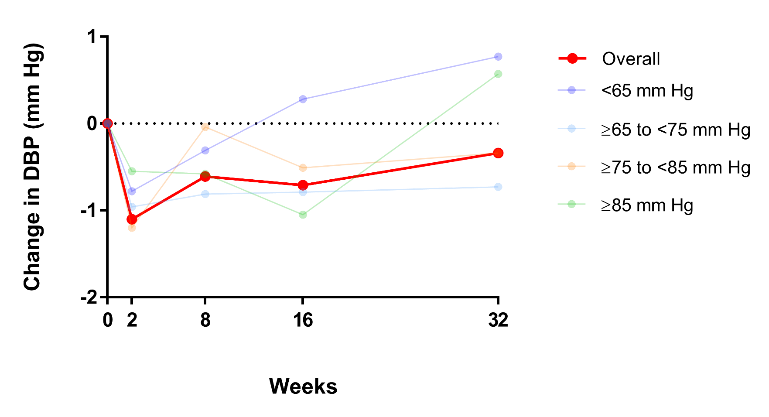


**FIGURE S3. Placebo-corrected change in pulse pressure with dapagliflozin from baseline to 2 weeks, 2 months, 4 months and 8 months.** The figure shows effect of dapagliflozin on pulse pressure during the first 8 months of treatment for the overall population and for each baseline pulse pressure groups.


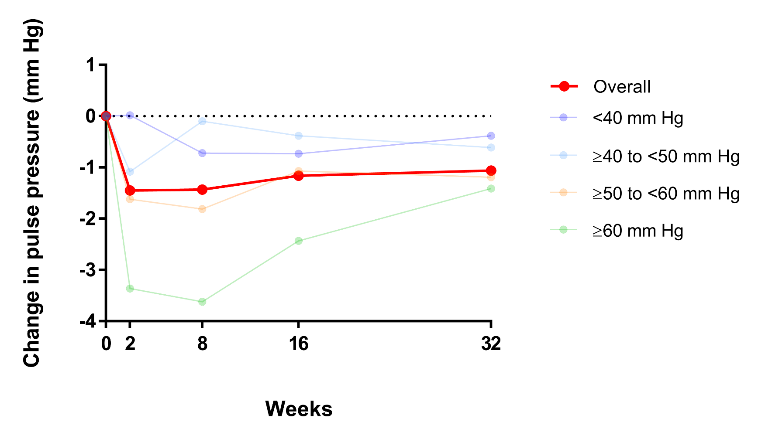


**Figure S4. Placebo-corrected change in blood pressure from baseline to two weeks with dapagliflozin, according to baseline LVEF.**

Blue dots represent mean blood pressure change, blue lines standard error.


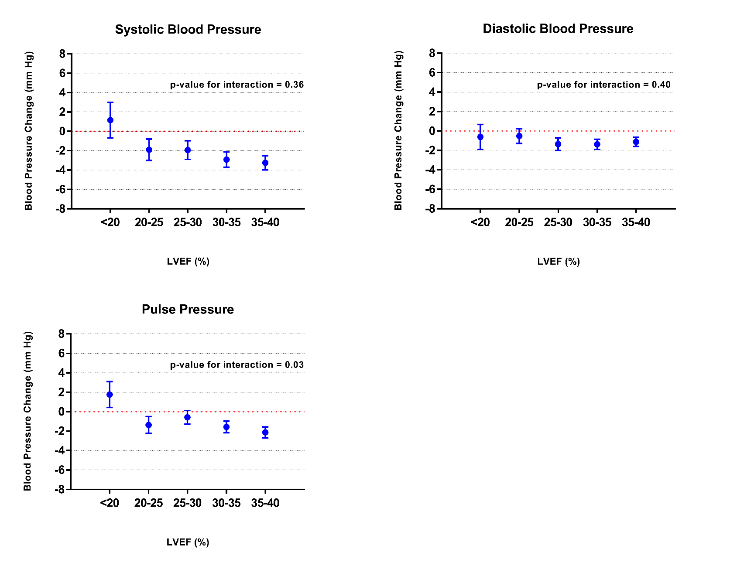


**FIGURE S5. Effect of Dapagliflozin on the Occurrence of Outcomes by Baseline Systolic Blood Pressure.** The figure shows adjusted hazard ratios for treatment with dapagliflozin compared to placebo according to baseline systolic blood pressure. The Cox regression model is adjusted for age, gender, race, region, heart rate, BMI, history of hypertension, type II diabetes, atrial fibrillation, previous MI, PCI, CABG, stroke, etiology of heart failure, baseline eGFR, ejection fraction, NYHA class and NT-proBNP level.
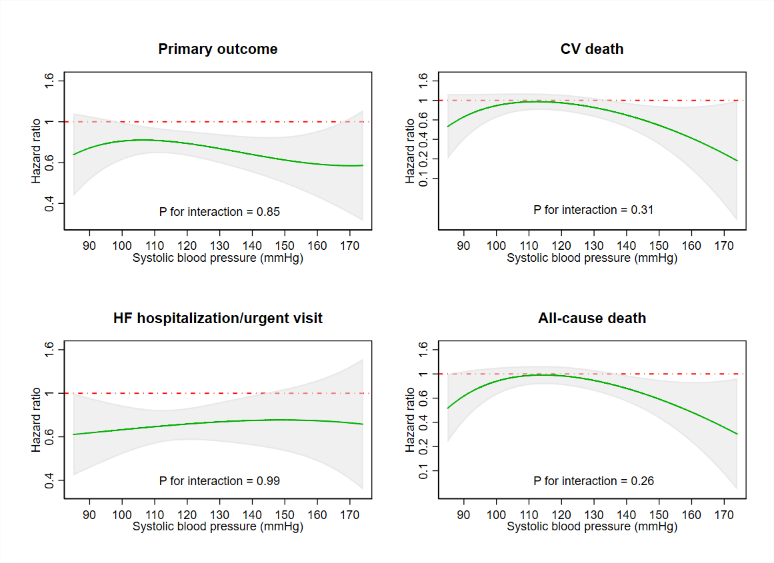


**FIGURE S6. Effect of dapagliflozin on the occurrence of outcomes by baseline diastolic blood pressure (DBP).** The figure shows unadjusted hazard ratios for treatment with dapagliflozin compared to placebo according to baseline diastolic blood pressure.


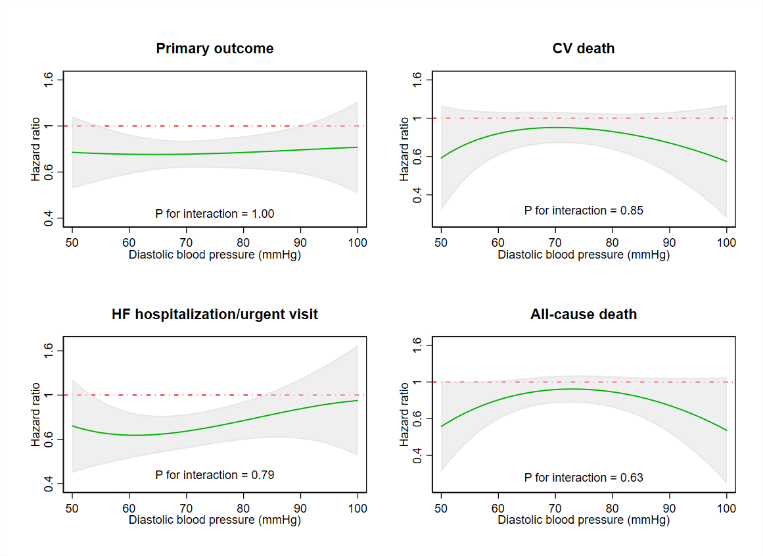


**FIGURE S7. Effect of dapagliflozin on the occurrence of outcomes by baseline pulse pressure.** The figure shows unadjusted hazard ratios for treatment with dapagliflozin compared to placebo according to baseline pulse pressure.


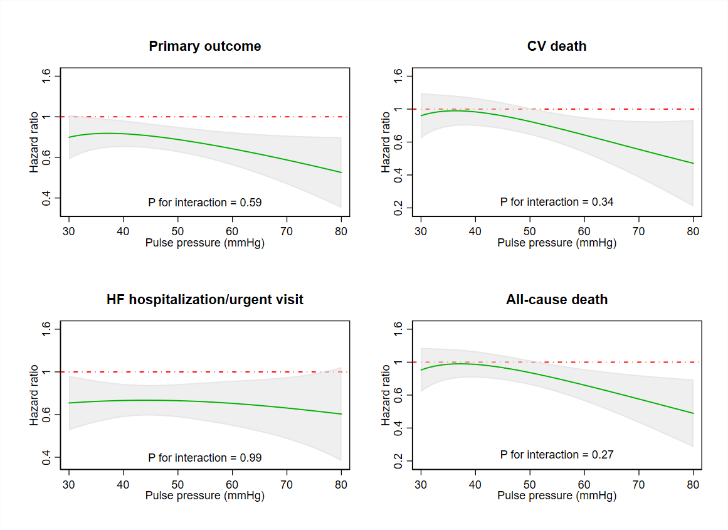


**FIGURE S8a. Effect of dapagliflozin on outcomes according to heart failure aetiology by systolic blood pressure.** The figure shows unadjusted hazard ratios for treatment with dapagliflozin compared to placebo according to baseline systolic blood pressure.


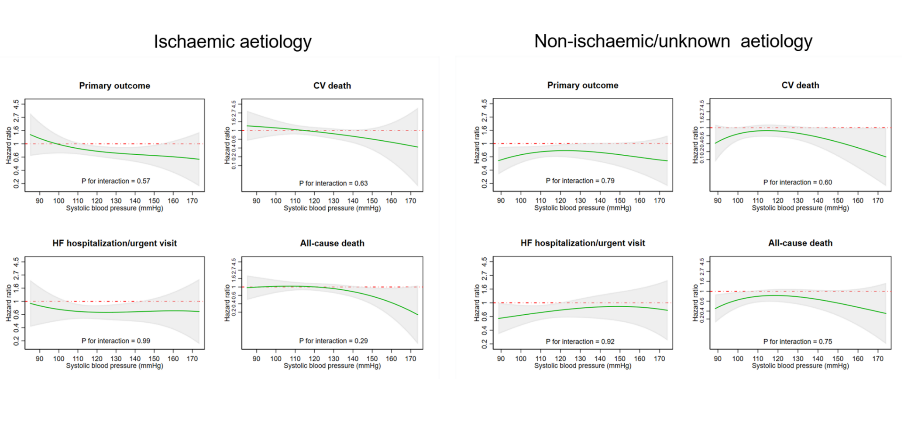


**FIGURE S8b. Effect of dapagliflozin on outcomes according to heart failure aetiology by diastolic blood pressure.** The figure shows unadjusted hazard ratios for treatment with dapagliflozin compared to placebo according to baseline diastolic blood pressure.
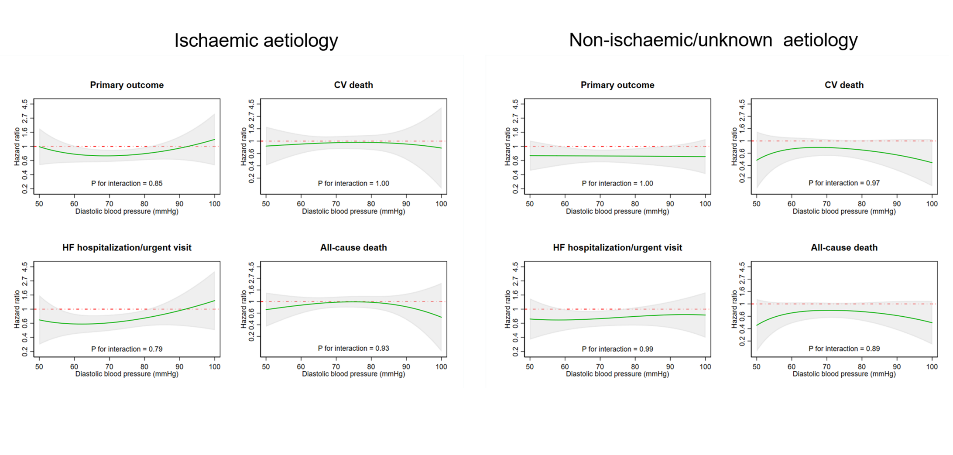


**FIGURE S9a. Effect of dapagliflozin on outcomes according to history of hypertension by systolic blood pressure.** The figure shows unadjusted hazard ratios for treatment with dapagliflozin compared to placebo according to baseline systolic blood pressure.
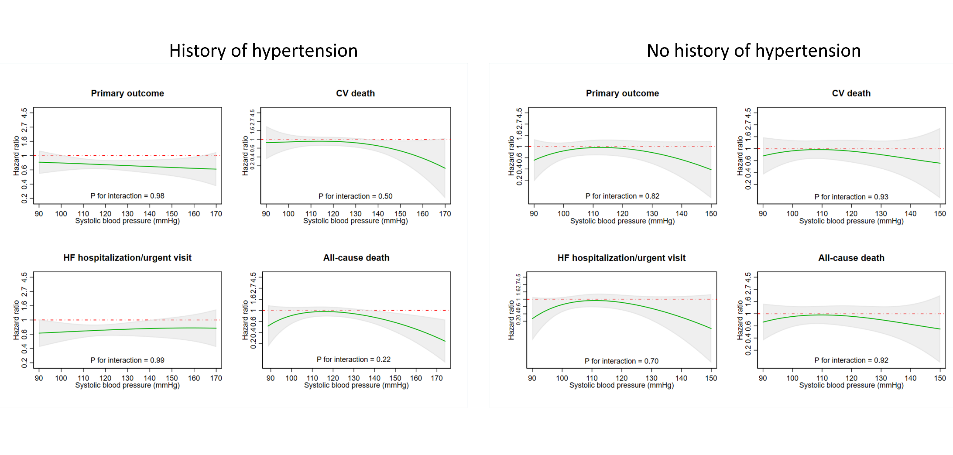


**FIGURE S9b. Effect of dapagliflozin on outcomes according to history of hypertension by diastolic blood pressure.** The figure shows unadjusted hazard ratios for treatment with dapagliflozin compared to placebo according to baseline diastolic blood pressure.
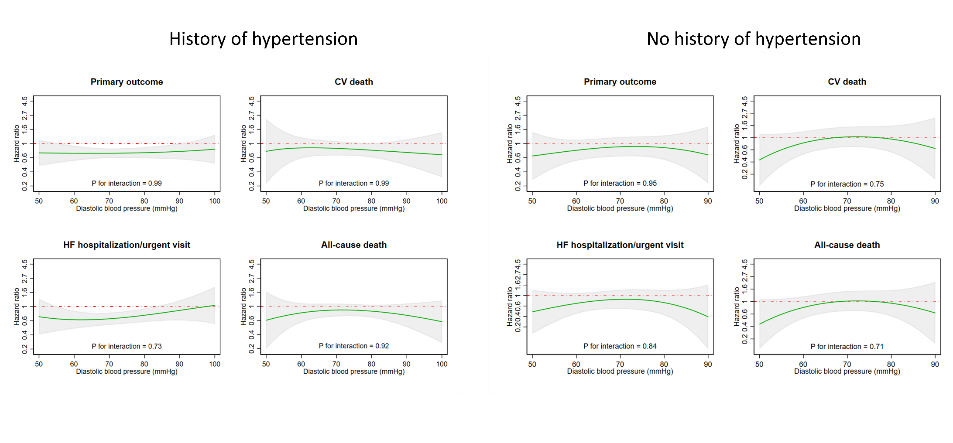


**FIGURE S10. Risk of clinical outcomes according to baseline systolic blood pressure.** The figure shows risk of primary endpoint (panel A), CV death (panel B) and HF hospitalization/urgent visit (panel C) for each treatment group and overall. An SBP of 130 mmHg is used as reference (Hazard ratio=1).

**
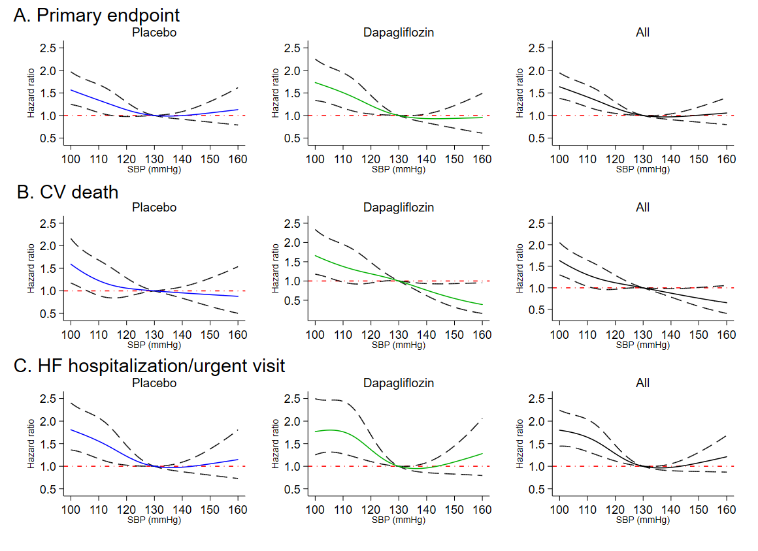
**

**FIGURE S11. Adjusted hazard ratios by treatment groups.** The figure shows risk for primary endpoint (panel A), CV death (panel B), HF hospitalization/urgent visit (panel C), All-cause death (panel D). The models are adjusted respectively for baseline SBP, baseline SBP category, time-updated SBP, time-updated SBP category. Placebo group is used as reference (Hazard ratio=1).

Dapa denotes dapagliflozin, CI confidence interval, HR hazard ratio, SBP systolic blood pressure.

**FIGURE S12. Risk of outcomes according to achieved SBP at 2 months.** Kaplan Meier event curves for the primary endpoint (A,B), cardiovascular death (C,D) and heart failure hospitalization/urgent visit (E,F) on placebo (left, A,C,E) or dapagliflozin (right, B,D,F) with a blood pressure performance above (orange, high) or below (green, low) 110 mmHg at 2 months.

**
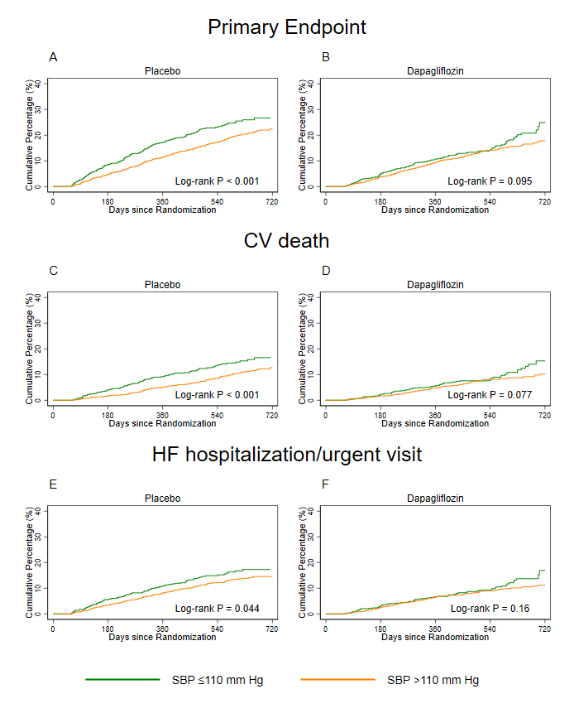
**

**FIGURE S13.** **Risk of outcomes according to change in SBP category from baseline to 2 months.** Kaplan Meier event curves for all cause death (A, B) and the primary endpoint (C, D) on placebo (A, C) or dapagliflozin (B, D) by low systolic blood pressure (SBP) at baseline [≤110 mmHg (low) versus >110 mmHg, high] and low or high SBP on treatment (at 2 months).


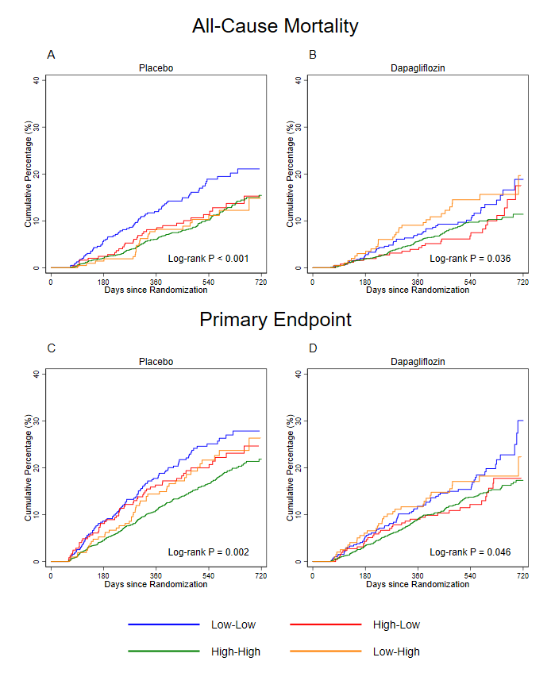

Supplement: ehaa496_Supplementary_Tables_and_Figures [file eurheartj_41_36_3402_s8.docx]
